# New insights into the genetic networks affecting seed fatty acid concentrations in *Brassica napus*

**DOI:** 10.1186/s12870-015-0475-8

**Published:** 2015-03-27

**Authors:** Xiaodong Wang, Yan Long, Yongtai Yin, Chunyu Zhang, Lu Gan, Liezhao Liu, Longjiang Yu, Jinling Meng, Maoteng Li

**Affiliations:** College of Life Science and Technology, Huazhong University of Science and Technology, Wuhan, 430074 China; National Key Lab of Crop Genetic Improvement, Huazhong Agricultural University, Wuhan, 430070 China; Institute of Biotechnology, Chinese Academy of Agricultural Sciences, Beijing, 100081 China; Key Laboratory of Cotton and Rapeseed, Ministry of Agriculture, Institute of Industrial Crops, Jiangsu Academy of Agricultural Sciences, Nanjing, 210014 China; College of Agronomy and Biotechnology, Southwest University, Chongqing, 400716 China

**Keywords:** *Brassica napus*, Fatty acid composition, QTL, Epistatic interaction, Regulatory pathway

## Abstract

**Background:**

Rapeseed (*B. napus*, AACC, 2n = 38) is one of the most important oil seed crops in the world, it is also one of the most common oil for production of biodiesel. Its oil is a mixture of various fatty acids and dissection of the genetic network for fatty acids biosynthesis is of great importance for improving seed quality.

**Results:**

The genetic basis of fatty acid biosynthesis in *B. napus* was investigated *via* quantitative trail locus (QTL) analysis using a doubled haploid (DH) population with 202 lines. A total of 72 individual QTLs and a large number pairs of epistatic interactions associated with the content of 10 different fatty acids were detected. A total of 234 homologous genes of *Arabidopsis thaliana* that are involved in fatty acid metabolism were found within the confidence intervals (CIs) of 47 QTLs. Among them, 47 and 15 genes homologous to those of *B. rapa* and *B. oleracea* were detected, respectively. After the QTL mapping, the epistatic and the candidate gene interaction analysis, a potential regulatory pathway controlling fatty acid biosynthesis in *B. napus* was constructed, including 50 enzymes encoded genes and five regulatory factors (*LEC1*, *LEC2*, *FUS3*, *WRI1* and *ABI3*). Subsequently, the interaction between these five regulatory factors and the genes involved in fatty acid metabolism were analyzed.

**Conclusions:**

In this study, a potential regulatory pathway controlling the fatty acid was constructed by QTL analysis and *in silico* mapping analysis. These results enriched our knowledge of QTLs for fatty acids metabolism and provided a new clue for genetic engineering fatty acids composition in *B. napus*.

**Electronic supplementary material:**

The online version of this article (doi:10.1186/s12870-015-0475-8) contains supplementary material, which is available to authorized users.

## Background

Oilseed rape (*Brassica napus* L., AACC, 2n = 38) is one of the most important oil crops producing multi-purpose oil for food and biofuel in many parts of the world. In 2007, biodiesel production accounted for 7% of the global vegetable oil supplies, in which 68% were used for biofuels in the EU [1]. As the global requirements for rapeseed oil are growing rapidly, increasing the oil content and improving the oil composition are important ways to meet the demands of agricultural feed stocks.

The fatty acid composition of rapeseed oil is considered to be genetically more variable than any other major vegetable oils [2]. Rapeseed oil is a mixture of seven main fatty acids [3]. Fatty acid biosynthetic pathways are generally controlled by multiple genes and considered as quantitative traits regulated by QTLs. So far, a number of QTLs controlling oil composition were identified in *B. napus.* Ecke *et al*. identified two QTLs for erucic acid distributed on chromosomes A6 and C2 [4]. Four QTLs for erucic acid distributed across chromosomes A1, A2, A8 and C3 were reported and three of these coincided with QTLs for the accumulation of oil content [5]. Burns *et al.* observed 13 QTLs affecting composition of 10 fatty acids, and seven also affected oil content [6]. Hu *et al.* identified two QTLs for oleic (on A1 and A5) and linolenic acids (on A4 and C4), respectively [7]. One to eight QTLs were detected for seven individual fatty acids by Zhao *et al*., and eight of these also affected oil content [8]. Recently, Smooker *et al*. identified 34 QTLs for five major fatty acids [9], and Yan *et al*. detected a total of 40 QTLs for six fatty acids, which were most clustered on chromosomes A8, A9 and C3 [10].

The allotetraploid *B. napus* has two progenitor species, *B. rapa* and *B. oleracea*, which shared their last common ancestor with *A. thaliana* about 20 million years ago [11,12]. Both a high degree of sequence similarities and chromosomal colinearities between *Brassica* species and *Arabidopsis* were reported [13-15]. Parkin *et al*. reported 21 conserved blocks within the *Arabidopsis* genome shared with *B. napus* [16], and Schranz *et al*. proposed a set of 24 conserved chromosomal blocks in *B. napus* [17]. Furthermore, all the genome sequence of *B. rapa*, *B. oleracea* and *B. napus* have been released [18-20]. It is feasible to predict the *Arabidopsis* orthologous genes for specific agronomic traits within the *Brassica* genome. For example, a number of candidate genes were mapped to CIs of QTLs for flowering time by *in silico* mapping [21], and a total of 14 lipid-related candidate gene loci were located in the CIs of six QTLs for seed oil content [22]. In fact, many important genes involved in fatty acid metabolism were identified in *Arabidopsis*, such as *FAB2*, *FAD2*, *FAD3* and *FAE1* [23-26], and the orthologs of these genes in *B. napus* were also reported and mapped. *BnaFAD2* was mapped on A1, A5, C1 and C5 chromosomes [27-29], and one major QTL *BnaA.FAD2.a* located on A5 was responsible for high C18:1 [27]. *BnaFAD3* was mapped on A3, A4, A5, C3 and C4 [9,27], and two major QTLs *BnaA.FAD3.b* and *BnaC.FAD3.b* were both responsible for low C18:3 [27]. *BnaFAE1* was mapped on both A8 and C3 [9], and two *FAE1* homologous genes on A8 and C3 linkage groups were also found by Qiu *et al*. [5] and Fourmann *et al*. [30]. Collectively, although genes or QTLs for fatty acid biosynthesis have been identified, the genetic network for all these metabolic pathways in *B. napus* needs to be elucidated.

In *Arabidopsis*, more than 120 enzymatic reactions and at least 600 genes are involved in acyl-lipid metabolism [31]. Li-Beisson *et al*. gave metabolic pathways associated with the biosynthesis and degradation of acyl-lipids in *Arabidopsis* [31]. The genome of polyploid *B. napus* may typically contain six distinct alleles for each gene present in *Arabidopsis* [32], the fatty acid biosynthesis and the gene regulation in *B. napus* might have a more complex pathway than that in *Arabidopsis*. Though much attention was given to genes and regulatory factors involved in acyl-lipid metabolism in *Arabidopsis* [31,33-37], similar questions concerning the genetic basis of fatty acid biosynthesis in *B. napus* remain open, mainly due to the lack of integrative studies at a population scale. Moreover, the interaction of genes involved in acyl-lipid metabolism has not yet been studied based on co-location of mapped candidate genes with QTLs in *B. napus*. To determine these key steps in relevant complex metabolic pathways of acyl-lipids in *B. napus*, it is first necessary to identify QTLs or genes associated with fatty acids composition.

In this paper, we describe the genetic bases of seed fatty acid composition through QTL mapping in *B. napus*. The aims of this study were as follows: (1) to add knowledge concerning QTL mapping of the fatty acid composition in *B. napus*; (2) to predict candidate genes of major QTLs for different fatty acids’ biosynthesis by comparative genome analysis; and (3) to construct a regulatory pathway for fatty acids metabolism in *B. napus*.

## Results

### Variation and single QTL analysis of fatty acid composition in the ‘Tapidor’ × ‘Ningyou7’ cross (TN) DH population

**Figure 1:**
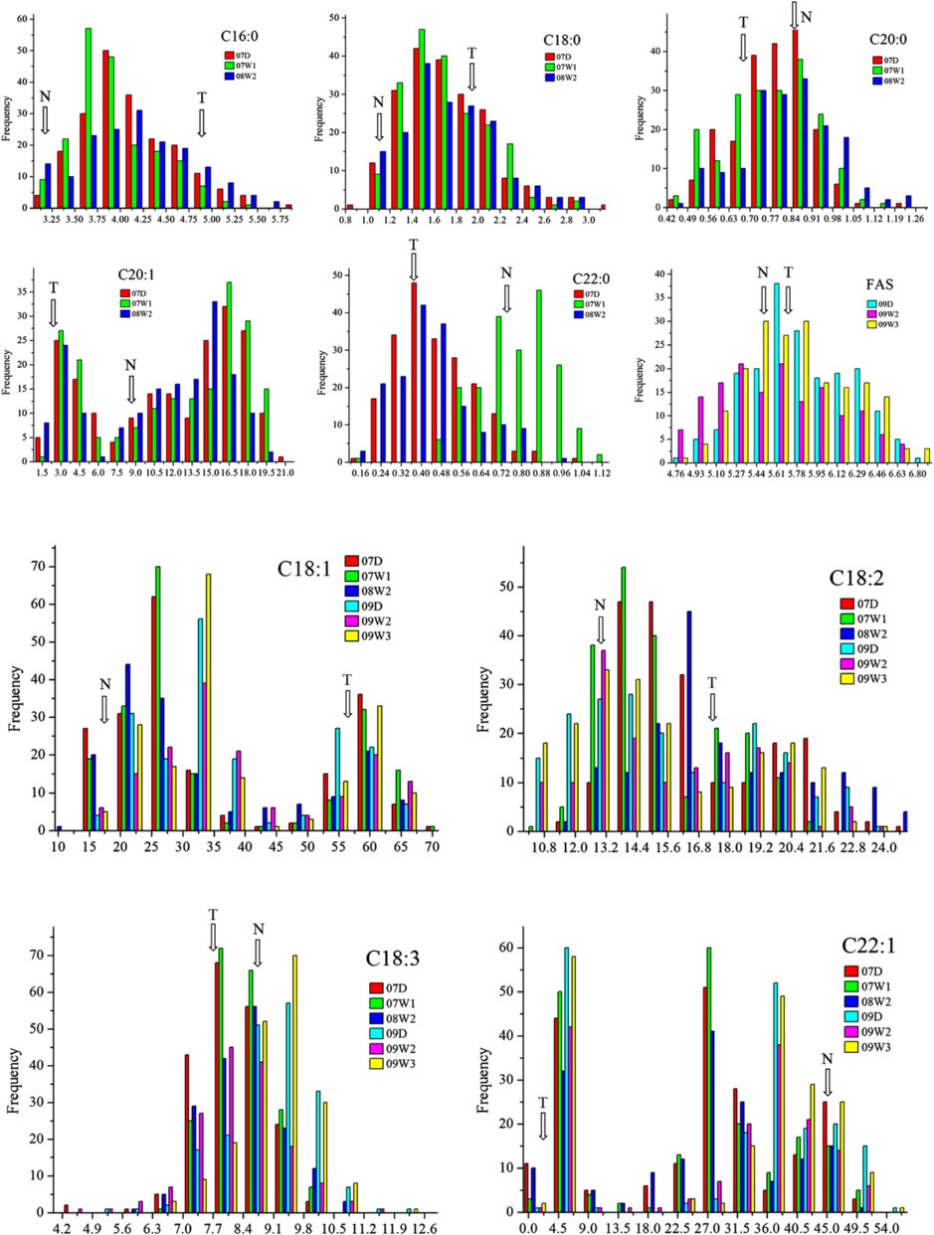
**Distribution of fatty acid concentrations of TN DH population in multiple environments.** The unit of x-axis means percentage of the specific fatty acid composition in the sum of all fatty acids. The unit of y-axis means the number of lines. N represents the parent “Ningyou7” and T the parent “Tapidor” of TN population.

Means of all traits measured from the TN DH population over six environments were close to the mid-parent values (Table 1). There was a wide range of variations and transgressive segregations for the concentration of each fatty acid (Figure 1). The population appeared to have a normal or near-normal distribution for C16:0, C18:0, C18:2, C18:3, C20:0, C22:0 and FAS (Saturated Fatty Acid), suggesting complexity of their genetic networks. However, C18:1, C20:1 and C22:1 showed bi-modal distributions, indicating that they might be controlled by few major genes with a relatively large effect. The distribution patterns of 10 fatty acids’ compositions showed that they were genetically stable but also affected by environment. The correlation between different fatty acid compositions showed great differences (Table 2). Erucic acid (C22:1) content was highly and positively correlated with the level of C20:0, C20:1 and C22:0 (Coefficients 0.30–0.63), but was negatively correlated with other fatty acids (−0.92 to −0.03), especially C18:1 (−0.92) (Table 2). C18:1 showed a high positive correlation with C16:0 and C18:0 (0.70–0.75) and moderate positive correlation with C18:2 and C18:3 (Table 2), but showed a high negative correlation with other fatty acids (−0.92 to −0.33).

**Table 1:** **Means and ranges for seed fatty acids of TN DH population evaluated in six environments**

**Traits**		**16:0**	**C18:0**	**C18:1**	**C18:2**	**C18:3**	**C20:0**	**C20:1**	**C22:0**	**C22:1**
Tapidor	Mean^a^	4.82 ± 0.03	1.89 ± 0.1	57.43 ± 2.46	17.5 ± 0.84	7.76 ± 0.52	0.67 ± 0.01	2.41 ± 1.24	0.35 ± 0.01	2.83 ± 0.91
Ningyou7	Mean	3.15 ± 0.01	1.11 ± 0.04	17.45 ± 2.85	12.92 ± 1.45	8.54 ± 0.48	0.85 ± 0.04	8.82 ± 0.08	0.73 ± 0.01	45.55 ± 0.27
DH	Mean	3.94 ± 0.53	1.63 ± 0.41	34.12 ± 15.63	15.44 ± 3.3	8.22 ± 0.97	0.77 ± 0.3	10.88 ± 5.62	0.4 ± 0.16	24.3 ± 15.56
	Max	5.87	4.92	73.54	27.24	12.02	6.88	19.66	1.86	52.22
	Min	2.84	0.89	9.4	8.61	1.31	0.14	1.08	0	0

^a^Mean value ± SE.

**Table 2:** **Pearson correlation coefficients for trait pairs affecting fatty acid compositions in the DH population**

	**C16:0**	**C18:0**	**C18:1**	**C18:2**	**C18:3**	**C20:0**	**C20:1**	**C22:0**	**C22:1**
C16:0	1								
C18:0	0.62^**^	1							
C18:1	0.70^**^	0.75^**^	1						
C18:2	0.06	0.05	0.03	1					
C18:3	0.26^**^	0.06	0.03	−0.01	1				
C20:0	−0.23^**^	−0.12^**^	−0.33^**^	−0.03	−0.13^**^	1			
C20:1	−0.58^**^	−0.52^**^	−0.77^**^	−0.03	−0.12^**^	0.31^**^	1		
C22:0	−0.37^**^	−0.41^**^	−0.51^**^	−0.04	−0.16^**^	0.27^**^	0.11^**^	1	
C22:1	−0.77^**^	−0.78^**^	−0.92^**^	−0.04	−0.03	0.30^**^	0.63^**^	0.60^**^	1

**Significant at *P* = 0.001.

**Figure 2:**
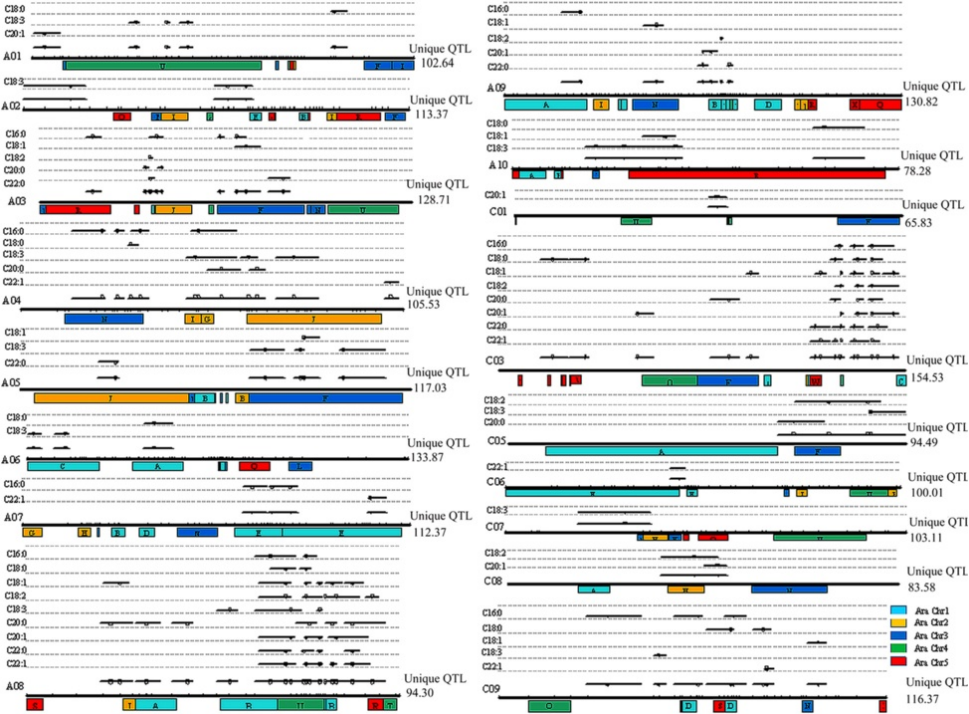
**QTL distribution of fatty acid concentrations on linkage groups in** ***B. napus*** **.** Whole linkage groups are shown with black lines labeled with molecular markers (short vertical bars) on the bottom, and the Arabic numerals listed on the right side show the length of linkage groups containing QTLs. The names of traits are listed on the left side of the linkage groups. The black lines on the linkage groups show the QTL confidence interval and the circles indicate the peak position. The pseudo-chromosomes of *Arabidopsis* are aligned under each linkage group of *B. napus*.

For QTL mapping analysis, Wincart_2.5 detected a total of 139 QTLs distributed across 15 chromosomes (except for C1, C2, C4 and C7) and individual QTL for any given trait explained 1.27–47.56% of phenotypic variance (PV) (Additional file 1). QTLNetwork_2.0 detected a total of 44 QTLs across 15 chromosomes (Additional file 2). After combining QTLs for different traits clustered in the same regions indicated by the same close-linked molecular markers, a total of 72 QTLs controlling fatty acid composition were identified (Figure 2, Table 3), and 10, 44 and 18 QTLs were detected by using QTLNetwork_2.0 only, Wincart_2.5 only and both QTLNetwork_2.0 and Wincart_2.5, respectively (Table 3).

**Table 3:** **The combined QTLs for fatty acid contents detected by WinQTLCart_2.5 and QTLNetwork_2.0**

**QTL**	**Chr.** ^**a**^	**Marker interval**	**LOD**	**Additive**	**PV(%)**	**range**	**S** ^**b**^	**Env.** ^**c**^	**traits**
***qA1-1***	**A1**	**znS13M26-100-CB10081**	**3.1**	**−1.28**	**4.87**	**0.3-7.2**	**W**	**07D**	**C20:1**
*qA1-2*	A1	CB10097-JICB0313	3.6	0.19	7.15	26.1-28.8	W	07 W1	C18:3
***qA1-3***	**A1**	**HBr006-ZAAS156a**	**7.6**	**0.29**	**14.64**	**35.9-37**	**W**	**07 W1**	**C18:3**
*qA1-4*	A1	JICB0455-znS08M15-320	4.8	0.24	11.42	39.8-43.1	W	07 W1	C18:3
***qA1-5***	**A1**	**em09me21-70-ZAAS165**	**4.0**	**0.07**	**3.43**	**80.4-84.6**	**W&Q**	**07 W1**	**C18:0**
***qA2-1***	**A2**	**CB10355-Ol10F04**	**3.1**	**0.23**	**6.64**	**0-18.2**	**W&Q**	**09 W3**	**C18:3**
***qA2-2***	**A2**	**HR-Sp1-210-pX154**	**3.3**	**0.31**	**7.85**	**56.2-61.5**	**W**	**09 W2**	**C18:3**
***qA2-3***	**A2**	**BRMS-082-HG-FT-A2**	**3.5**	**0.32**	**8.43**	**61.5-67.3**	**W**	**09 W2**	**C18:3**
*qA3-1*	A3	ZNS13M26-360-HBR160		0.05	2.02	15.7-21.4	Q		C16:0
***qA3-2***	**A3**	**RA2E11-BRMS-303**	**3.8**	**−0.03**	**4.02**	**35.9-37.7**	**W**	**07 W1**	**C20:0**
*qA3-3*	A3	CB10271-CNU250	3.3-4.0	−0.52-0.02	3.01-5.66	37.8-43.0	W&Q	07D/07 W1	C16:0/C18:2/C20:0/C22:0
*qA3-4*	A3	HBr124-KBrB043L02-12	3.1	−0.10	3.22	61.5-63.7	W	07D	C16:0
*qA3-5*	A3	HBr137-CNU270	3.0-3.5	−0.1-3.2	3.52-3.56	67.4-76.1	W&Q	07D/08 W2	C16:0/C18:1
*qA3-6*	A3	HR-S2-130-HR-Tp4-165	3.8	−0.03	4.78	79.0-86.5	W	07D	C22:0
***qA4-1***	**A4**	**OL11H02C-SR12307I**		**−1.02**	**0.39**	**100.1-104.1**	**Q**		**C22:1**
*qA4-2*	A4	znS13M26-220-sN3514f	4.4	0.18	8.86	14.0-23.2	W	08 W2	C16:0
*qA4-3*	A4	IGF3365B-SN13034		−0.09	5.43	25.7-28.5	Q		C16:0
***qA4-4***	**A4**	**BRMS-276-HR-C001-A4**	**4.6**	**0.06-0.17**	**1.04-8.02**	**29.5-35.2**	**W&Q**	**08 W2**	**C16:0/C18:0**
*qA4-5*	A4	niab048-JICB0134	3.4-5.4	−0.03-0.18	5.45-6.26	45.6-60.5	W	07D/07 W1	C16:0/C18:3/C20:0
*qA4-6*	A4	JICB0134-HBr091	5.22	−0.05_-0.04	0.37-5.44	60.5-67.2	W&Q	07 W1	C18:3/C20:0
*qA4-7*	A4	HBr202-HS-k02-2	3.2	0.24	6.98	70.4-82.3	W	07D	C18:3
***qA5-1***	**A5**	**BRMS-034-niab017**	**3.3**	**0.03**	**4.18**	**22.9-29.3**	**W**	**07D**	**C22:0**
*qA5-2*	A5	niab013-pX129a	3.8	0.09	4.40	57.9-59.9	W	09D	FAS
*qA5-3*	A5	CNU206-PIE1-6	3.2-3.7	0.09-0.26	4.38-8.17	65.3-79.0	W&Q	09 W3/09D	C18:3/FAS
*qA5-4*	A5	CNU268-HR-Tp4-200	3.1-3.3	−2.3-0.22	1.88-5.96	82.3-89.7	W	07D	C18:1/C18:3
*qA5-5*	A5	IGF3134a-CNU362	3.8	0.23	6.76	95.5-109.3	W	07D	C18:3
*qA6-1*	A6	Ol11F12a-ZAAS92a	4.8	0.29	10.56	0-4.6	W&Q	07D	C18:3
***qA6-2***	**A6**	**HBr201-BRMS-030**	**3.2**	**−0.07**	**3.05**	**40.7-51.1**	**W&Q**	**07D/07 W1**	**C18:0**
***qA6-3***	**A6**	**HR-Tp3-320-pW217**	**4.1**	**0.25**	**7.51**	**9.1-14.2**	**W**	**07D**	**C18:3**
***qA7-1***	**A7**	**IGF1226l-CNU053b**	**3**	**−1.70**	**1.27**	**100.2-105.5**	**W**	**07D**	**C22:1**
*qA7-2*	A7	HBr030-znS06M34-50	5.4	−0.10	4.36	64.0-70.7	W	07 W1	C16:0
***qA7-3***	**A7**	**CNU167-CNU044**	**4.4**	**−0.09**	**3.62**	**71.2-74.8**	**W**	**07 W1**	**C16:0**
***qA7-4***	**A7**	**BRMS-036-HBr021**	**4.5**	**−0.10**	**4.10**	**74.8-79.8**	**W**	**07 W1**	**C16:0**
*qA8-1*	A8	em10me26-120-JICB0335		−0.04-0.64	4.39-6.42	18.6-26.8	Q		C18:1/C20:0
*qA8-2*	A8	HBr107-HBr017	8.1	0.07	15.24	27.4-34.0	W	08 W2	C20:0
***qA8-3***	**A8**	**HBr104-HBr010**	**4.7**	**0.05**	**9.30**	**36.8-41.9**	**W**	**08 W2**	**C20:0**
*qA8-4*	A8	Na12B05a-HBr031	3.4	−0.22	6.16	48.0-53.3	W	07D	C18:3
***qA8-5***	**A8**	**IGF1108c-sR7178**	**3.1-59.9**	**−10.4-9.14**	**5.7-47.55**	**57.4-74.6**	**W&Q**	**07D/07 W1/08 W2/09D/09 W2/09 W3**	**C16:0/C18:0/C18:1/C18:2/C18:3/C20:0/C20:1/C22:0/C22:1/FAS**
*qA8-6*	A8	HBr015-HBr026	3.9-33.2	−9.1-8.9	7.7-38.7	75.3-90.4	W	07D/07 W1/08 W2/09 W2	C18:1/C18:2/C20:0/C20:1/C22:0/C22:1/FAS
*qA9-1*	A9	pW123aH-HBr178	3.6	0.08	2.74	18.3-25.3	W&Q	07 W1	C16:0
*qA9-2*	A9	CNU296-KBrB073D09-12	3.2	−3.20	4.64	45.5-52.0	W	09 W2	C18:1
***qA9-3***	**A9**	**HBr197-HBr205**	**4.5**	**0.04**	**5.59**	**63.5-66.9**	**W**	**07D**	**C20:1**
*qA9-4*	A9	HBr205-niab003	4.3	−1.50	6.47	66.9-70.0	W&Q	07 W1	C20:1
*qA9-5*	A9	IGF1087g-niab127	3.3	0.52	3.32	70.9-71.5	W	07D	C18:2
*qA9-6*	A9	em18me23-380-HBr196	3.9	0.03	4.97	73.2-75.1	W	07D	C22:0
*qA9-7*	A9	pW203b-HBr053	3.4	−0.08	3.96	86.1-95.5	W	09D	FAS
*qA10-1*	A10	RA2E03-HS-b14-1	3.5	0.24	6.29	14.6-18.9	W	07D	C18:3
*qA10-2*	A10	HS-j90-HG4-HG-CO-A10	3.3-4.6	−2.59-0.42	2.0-12.9	18.9-34.2	W&Q	07D/07 W1/09 W2	C18:0/C18:1/C18:3
*qA10-3*	A10	JICB0551-JICB0573		−0.05	0.28	60.8-71.3	Q		C18:0
***qC1-1***	**C1**	**EM18ME23-300-CB10258**		**0.58**	**3.85**	**33.0-35.9**	**Q**		**C20:1**
*qC3-1*	C3	IGF5376b-HBr014	3.4-15.1	−8.20-8.63	5.0-30.0	118.4-126.1	W	07D/09D	C18:1/C22:0/C22:1
*qC3-2*	C3	HBr014-Ol13C12	4.7-38.6	−9.83-8.86	6.3-37.7	126.1-131.5	W	07D/07 W1/08 W2/09D/09 W2/09 W3	C16:0/C18:0/C18:1/C18:2/C20:0/C20:1/C22:0/C22:1/FAS
*qC3-3*	C3	IGF0235b-BRMS-093	3.8-64.9	−10.96-9.78	3.0-45.8	133.8-152.3	W&Q	07D/07 W1/08 W2/09D/09 W2/09 W3	C16:0/C18:0/C18:1/C18:2/C20:0/C20:1/C22:0/C22:1/FAS
*qC3-4*	C3	pX141bE-HBr032	4.9	0.10	4.80	15.7-26.0	W&Q	07D	C18:0
*qC3-5*	C3	pW221-pX141aE	4.4	0.07	3.68	26.5-34.2	W	07D/07 W1	C18:0
*qC3-6*	C3	HBr211-HBr152	4.2	1.52	6.46	51.7-59.0	W	07 W1	C20:1
*qC3-7*	C3	Ol11G11b-pW143		−0.05	4.98	80.3-91.5	Q		C20:0
*qC3-8*	C3	HBr139-CNU099	4.7	−3.10	2.91	94.1-98.8	W	07D	C18:1
***qC5-3***	**C5**	**Ol10B02-JICB0509**	**3.2**	**0.10**	**5.01**	**30-62.6**	**W**	**09 W3**	**FAS**
*qC5-1*	C5	IGF3112a-em12me21-150	3.5-7.2	−0.06-0.55	3.7-13.9	63.8-82.9	W	07D/08 W2	C18:2/C20:0
***qC5-2***	**C5**	**em12me21-150-IGF0193C**	**4.2-4.6**	**0.28-0.56**	**3.9-9.8**	**82.9-88.3**	**W**	**07D/09 W3**	**C18:2/C18:3**
*qC6-1*	C6	HBr025-Na12E01a	5.5	0.24	11.4	74.3-80.8	W	09 W2	FAS
***qC6-2***	**C6**	**HBr057-HBr047**	**4.0**	**−0.21**	**8.64**	**91.0-95.4**	**W**	**09 W2**	**FAS**
***qC6-3***	**C6**	**SR12387-EM14ME28-200**		**−0.57**	**0.10**	**41.2-45.3**	**Q**		**C22:1**
*qC7-1*	C7	SNRH63-CNU400		−0.13	1.68	18.8-37.2	Q		C18:3
*qC8-1*	C8	CB10504-sN11670a	3.4	0.36-0.54	3.0-3.65	32.4-46.8	W&Q	07D/09 W3	C18:2/C20:1
***qC9-1***	**C9**	**CB10064-em20me27-230**	**6.1**	**−0.12**	**6.28**	**26.3-42.9**	**W**	**07 W1**	**C16:0**
***qC9-2***	**C9**	**EM20ME27-230-HR-TP3-360**		**0.13**	**3.83**	**46.2-50.3**	**Q**		**C18:3**
*qC9-3*	C9	BRMS-154-HBr144	5	−0.12	5.31	52.7-61.2	W&Q	07D/07 W1	C16:0
*qC9-4*	C9	HBr186-SA30	3.1-4.1	−0.14-0.08	3.97-5.4	62.5-74.2	W	07D/08 W2	C16:0/C18:0
***qC9-5***	**C9**	**S16071-3F3R-sNRG42**	**3.4**	**0.07-0.90**	**0.03-3.3**	**76.2-82.8**	**W&Q**	**07D**	**C18:0/C22:1**
*qC9-6*	C9	HR-Sp1-170-sR12384I	3.6	2.71	3.36	92.7-98.4	W	09 W3	C18:1

^a^Chromosome.

^b^The software used to detect QTL. W, WinQTLCart_2.5; Q, QTLNetwork_2.0.

^c^The environment in which the QTLs are detected.

The QTL with bold indicates that no candidate genes are located in the confidence interval of this QTL in the present study.

Individual saturated fatty acids were only analyzed in three environments (07D, 07 W1 and 08 W2). For C16:0, 18 QTLs were detected across seven chromosomes (Table 3). Among these QTLs, seven (39%) were detected by two types of software. The additive effect ranged from −0.29 to 1.72, and explained 2.74–37.99% of PV (Additional files 1 and 2). For C18:0, 12 QTLs distributed across seven chromosomes had −0.24 to 0.10 additive effect and explained 3.05–37.73% of PV (Table 3, Additional files 1 and 2). Three of them (on A1, A4 and C3) were environment-specific QTLs detected only in one environment. A total of 13 QTLs controlling C20:0 explained 4.02–47.55% of PV (Table 3, Additional files 1 and 2). Nine QTLs on five chromosomes for C22:0 were detected, and three were environment-specific, their additive effect ranged from −0.06 to 0.11, and explained 4.02–47.56% of PV (Table 3, Additional files 1 and 2). In 09D, 09 W2 and 09 W3, all saturated fatty acid compositions were considered as one trait named FAS, and a total of 10 QTLs were detected with additive effect ranged from −0.28 to 0.24 and explaining 3.96–31.33% of PV (Table 3, Additional files 1 and 2).

For the other four unsaturated fatty acid components, phenotypic data were obtained from six different environments, except for C20:1 from three environments (07D, 07 W1 and 08 W2). Twelve QTLs for C18:1 were distributed across seven chromosomes and their additive effect ranged from −10.96 to 3.18 and explained 1.88–42.43% of PV (Table 3, Additional files 1 and 2). Nine QTLs on six chromosomes were associated with C18:2, with the additive effect ranging from −1.69 to 0.56 and explaining 3.10–36.26% of PV (Table 3, Additional files 1 and 2). Twenty-one QTLs on 10 chromosomes were significantly associated with C18:3, which had −1.64 to 0.42 additive effect and explained 5.70–22.29% of PV (Table 3, Additional files 1 and 2). For C20:1, 10 QTLs were detected across A1, A8, A9, C1, C3 and C8 in three environments (Table 3). Six of them were found in two or more environments, and the remaining four were only in one environment. Nine QTLs were observed for C22:1 distributed across A4, A7, A8, C3, C6 and C9, and singly explained 1.27–45.79% of PV (Table 3, Additional files 1 and 2).

### Co-localization of mapped candidate genes of *B. rapa* and *B. oleracea* with single-locus QTL

**Figure 3:**
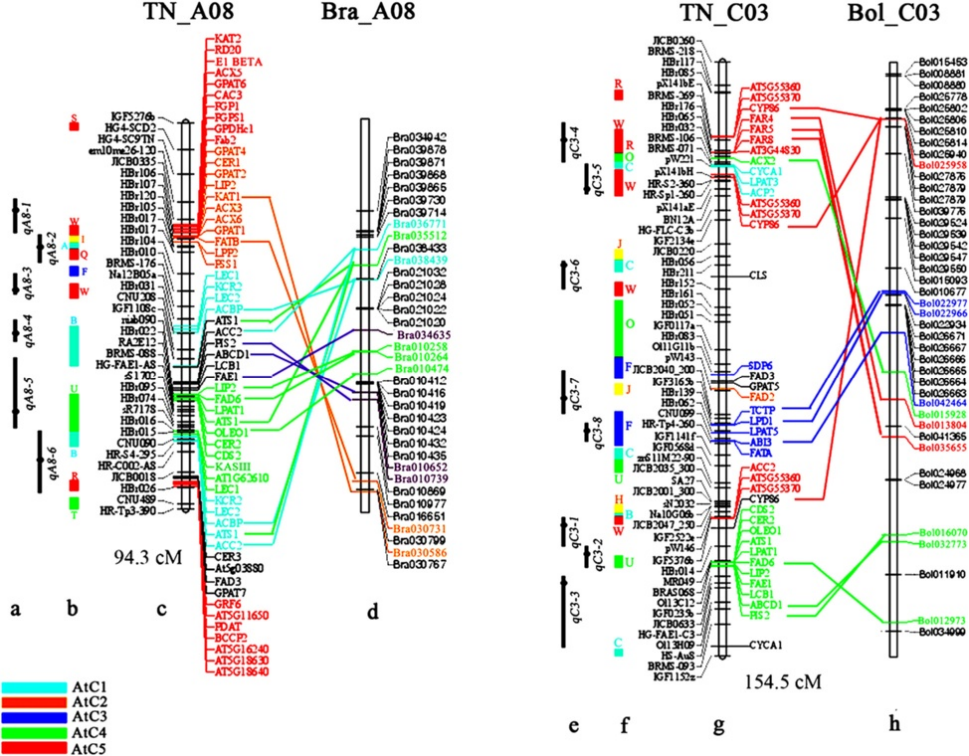
**Comparative mapping of homologous linkage groups between** ***B. napus*** **and** ***B. rapa/B. oleracea*** **. (a, e)** QTLs associated with 10 traits in multiple experiments, and the ring point in the bar indicates the QTL peak position. **(b, f)** The building blocks of *Brassica* genomes are identified on TN genetic maps. **(c, g)** On the left side are the markers on TN chromosome, and on the right side are candidate genes of *Arabidopsis* underlying the QTL confidence interval. **(d, h)** Genes with colored font are the candidate genes in *B. rapa/B. oleracea* of *Arabidopsis*, and genes with black font are the candidate genes in *B. rapa/B. oleracea* identified by each informative marker. The homologous genes in A/C genomes of *B. rapa/B. oleracea* and *B. napus* are connected with colored lines using the homologous genes as anchors. The genetic length of TN chromosome is annotated at the bottom of chromosome. The color-coding of the blocks is based on the report of Schranz *et al*. (2006) [66] and the candidate genes with the same color as the block.

A total of 932 molecular markers were mapped to the new version of the TN map. This map covered a total length of 2116.73 cM with an average marker interval of 2.27 cM. The length of the 19 linkage groups varied from 65.83 (C1) to 154.53 (C3) cM (Additional file 3). Thirty-four synteny blocks (28 for A genome, 6 for C genome) and 149 insertion fragment islands (95 for A genome, 54 for C genome) were identified between *Arabidopsis* pseudochromosomes and TN DH genetic linkage groups by the *in silico* mapping approach (Additional file 3). More than twenty-nine thousand homologous genes were found to underline the CIs of 61 QTLs associated with the concentrations of 10 different fatty acids, by comparative analysis between the linkage map of TN DH and the genome of *Arabidopsis* (Additional file 4). Among them, a total of 111 key genes involving fatty acid metabolism were used as candidate genes in the present study (Additional file 4). A number of important genes were found, such as *FAB2*, *FAD2*, *FAD3* and *FAE1*, but also five regulatory factors, *LEC1*, *LEC2*, *FUS3*, *WRI1* and *ABI3*. As the polyploid *B. napus* genome may typically contain six loci for each gene present in *Arabidopsis*, these 111 genes were found to have 824 homologous genes mapped on the TN DH linkage map in total, including 97 key genes of 234 homologs underlying the CIs of 47 QTLs (Additional file 4). All of the QTLs with CIs containing homologous genes were separately compared to the physical genomic regions of *B. rapa* (A genome) and *B. oleracea* (C genome). To compare with the *B. rapa* genome, 32 QTLs containing candidate gene(s) distributed on the A genome of the TN DH linkage map were used for analysis, accounting for 65.3% of the total QTLs on the A genome. In total, 47 genes in *B. rapa* matched those in *Arabidopsis* underlying 24 QTL CIs (Additional file 4). For the C genome of the TN DH linkage map, 23 QTLs were detected and 15 (65.2%) of them contained candidate gene(s) in *Arabidopsis*. Comparison of the candidate genes showed 15 genes in *B. oleracea* matched those in *Arabidopsis* underlying 7 QTL CIs (Additional file 4). For example, on the C3 linkage group, 33 candidate genes (40 homologous genes) of *Arabidopsis* were located in the CIs of eight QTLs, and 10 candidate genes of *B. oleracea* were found based on the candidate genes of *Arabidopsis*, including the regulatory factor *ABI3* of previous studies (Figure 3).

### Epistatic QTLs and interaction analysis of candidate genes for fatty acid compositions

QTLNetwork_2.0 and Genotype Matrix Mapping ver2.1 (GMM) software were used to identify the epistatic interactions for fatty acid compositions. Twenty-four pairs of epistatic QTLs involving 28 loci were identified by QTLNetwork_2.0 for 10 measured traits (Additional files 5 and 6), with 1–5 epistatic QTL pairs for each trait. The proportion of total PV explained by all epistatic QTLs was 1.03–38.26% for each trait. Among 24 epistatic QTLs, two, three and 19 were NN, AN and AA interactions, respectively. A total of 395 loci interactions were identified by GMM, including 34 pairs of digenic and 361 pairs of trigenic interactions (Additional file 7). By comparing the epistatic interactions by GMM and single QTL based on common markers, 312 pairs of epistatic interactions were associated with QTLs. Some of the epistatic QTLs affected the level of more than one fatty acid composition. For example, the interactions of two loci associated with QTLs *qA8-5* and *qC3-3* were detected by both types of software, which controlled different fatty acid compositions (C16:0, C18:0, C18:1, C20:1, C22:0 and FAS by QTLNerwork_2.0 and nine traits except FAS by GMM; Additional files 6 and 7). This indicated that epistatic interactions were very important for fatty acid metabolism in *B. napus*.

For C16:0, four pairs of epistatic QTLs were detected by QTLNetwork_2.0, explaining 0.66–4.99% of PV. The interaction between QTL *qA8-5* and *qC9-3* explained 3.39% of PV (Additional file 6). The genes *OLEO1*, *ACC2*, *FAE1*, *LPAT1* and *ATS1* were underlying the QTL CI of *qA8-5*, while *LEC1*, *LEC2*, *ACC2*, *TAG1*, *KASIII* and *ATS1* were underlying the CI of *qC9-3* (Additional file 4). A total of 34 significant loci interactions were identified for C16:0 by GMM, containing one pair with digenic and 33 pairs with trigenic interactions, most of these trigenic interactions have loci underlying three QTLs of CIs, including QTLs *qA8-5*, *qA8-6* and *qC3-3* (Additional file 7). A number of homologous genes were mapped to the CI of *qA8-6*, including *LEC1*, *LEC2*, *ACC2*, *KASIII*, *FAD3*, *GPAT7*, *BCCP2*, *ACBP*, *PDAT*, and *ATS1*, but only one homologous gene *CYCA1* for *qC3-3* was found (Additional file 7). All the results above suggested that the potential of these gene interactions increased the level of C16:0. Similar to C16:0, a series of pairs of epistatic QTLs and loci interactions for another nine traits were identified by QTLNetwork_2.0 (Additional file 6) and GMM (Additional file 7), and numerous important genes underlying QTL CIs were identified (Additional file 4). For example, for C18:0, C18:1 and C18:2, besides the trigenic interaction of QTLs *qA8-5*, *qA8-6* and *qC3-3*, the interactions between QTLs *qC3-2* and *qA8-5* were also identified, and *FAE1* was underlying the CI of *qC3-2*.

**Figure 4:**
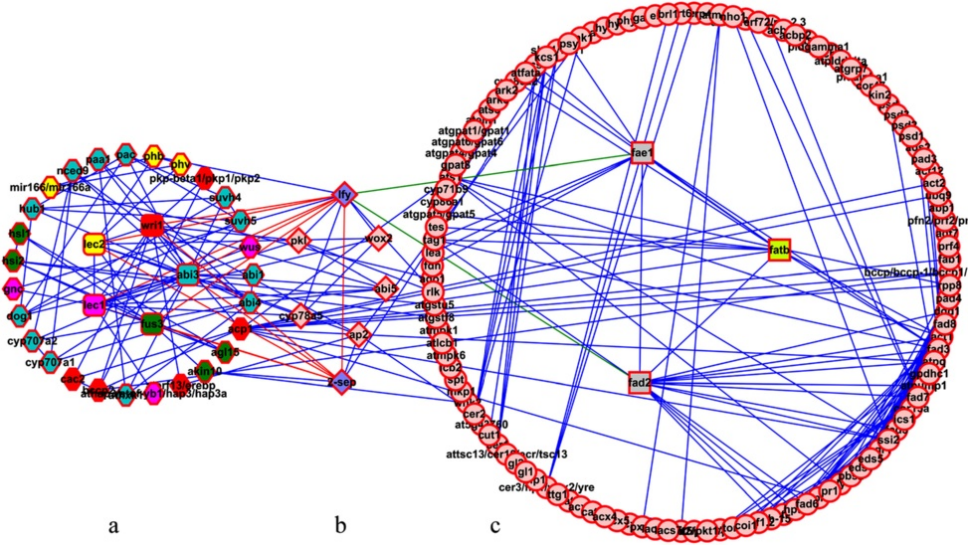
**Gene interaction pathway.** Network visualization for interaction of the 93 candidate genes and regulatory factors observed from the QTL results using Cytoscape_V2.6.3 software. Genes are presented as nodes and gene interactions are presented as edges. **(a)** The five regulatory factors and genes that are directly affected by only one regulatory factor: the five regulatory factors indicated by round rectangle with different color (yellow, *LEC2*; pink, *LEC1*; green, *FUS3*; light blue, *ABI3*; red, *WRI1*), while the genes are depicted as hexagon with the same color of the regulatory factors which linked to them. **(b)** The genes affected by two or more regulatory factors, denoting as diamond-shaped nodes, and the two diamond-shaped nodes with light blue color are *LFY* and *SEP2*, separately. **(c)** The genes indirectly affected by regulatory factors. In the middle of the cluster are three very important genes in fatty acid synthesis denoted as rectangle nodes with different color (pink, *FAD2*; green, *FAE1*; light yellow, *FATB*), and other genes are depicted as ellipse pink-filled nodes.

Cytoscape_V2.6.3 software was used to investigate the interaction of candidate genes that were observed from the single and epistatic QTL results and the gene interaction network was constructed. The results revealed that the whole network incorporated 167 nodes and 416 edges, that could be divided into three sub-clusters: the five regulatory factors (*FUS3*, *ABI3*, *WRI1*, *LEC1* and *LEC2*) and the genes that were directly affected by only one regulatory factor (A cluster, Figure 4a), the genes affected by two or more regulatory factors (B cluster, Figure 4b) and the genes indirectly affected by regulatory factors (C cluster, Figure 4c). The A cluster consisted of 32 nodes and 105 edges in total. The five regulatory factors formed a tightly intra-linked group, with each regulatory factor under the influence of at least two other regulatory factors, and *LEC1* and *LEC2* were especially affected by all other four regulatory factors. In addition to the five regulatory factors, *BCCP2* and *CAC2* were underlying the QTL CIs. *BCCP2* was associated with QTLs *qA3-1* and *qA8-6*, which affected C16:0, C18:2 and C20:0; *CAC2* was associated with QTL *qC9-4* and affected C18:0. These two genes were both regulated by *WRI1*. The B cluster was composed of seven nodes and 40 edges, in which the genes were affected by at least two regulatory factors. *SEP2* and *LFY* in this group were directly affected by all five regulatory factors. *LFY* was linked to other eight genes (Figure 4), and two of them (*FAD2* and *FAE1* in cluster C) were well-known important genes involved in fatty acid synthesis. The C cluster included 128 nodes and 319 edges, and 27 genes were detected to underline the QTL CIs. *FATB* associated with QTL *qA8-2* was notably affected by both *FAE1* and *FAD2*, and *FATB* also affected another eight genes (*ACP1*, *FAD3*, *FATA*, *ACT1*, *TES*, *TAG1*, *CYP86A2* and *GPAT5*) that were underlying the QTL CIs. The network mentioned above indicated that the genes involved in fatty acid synthesis were directly or indirectly affected by regulatory factors.

### Construct of a potential controlling pathway of fatty acids in *B. napus*

**Figure 5:**
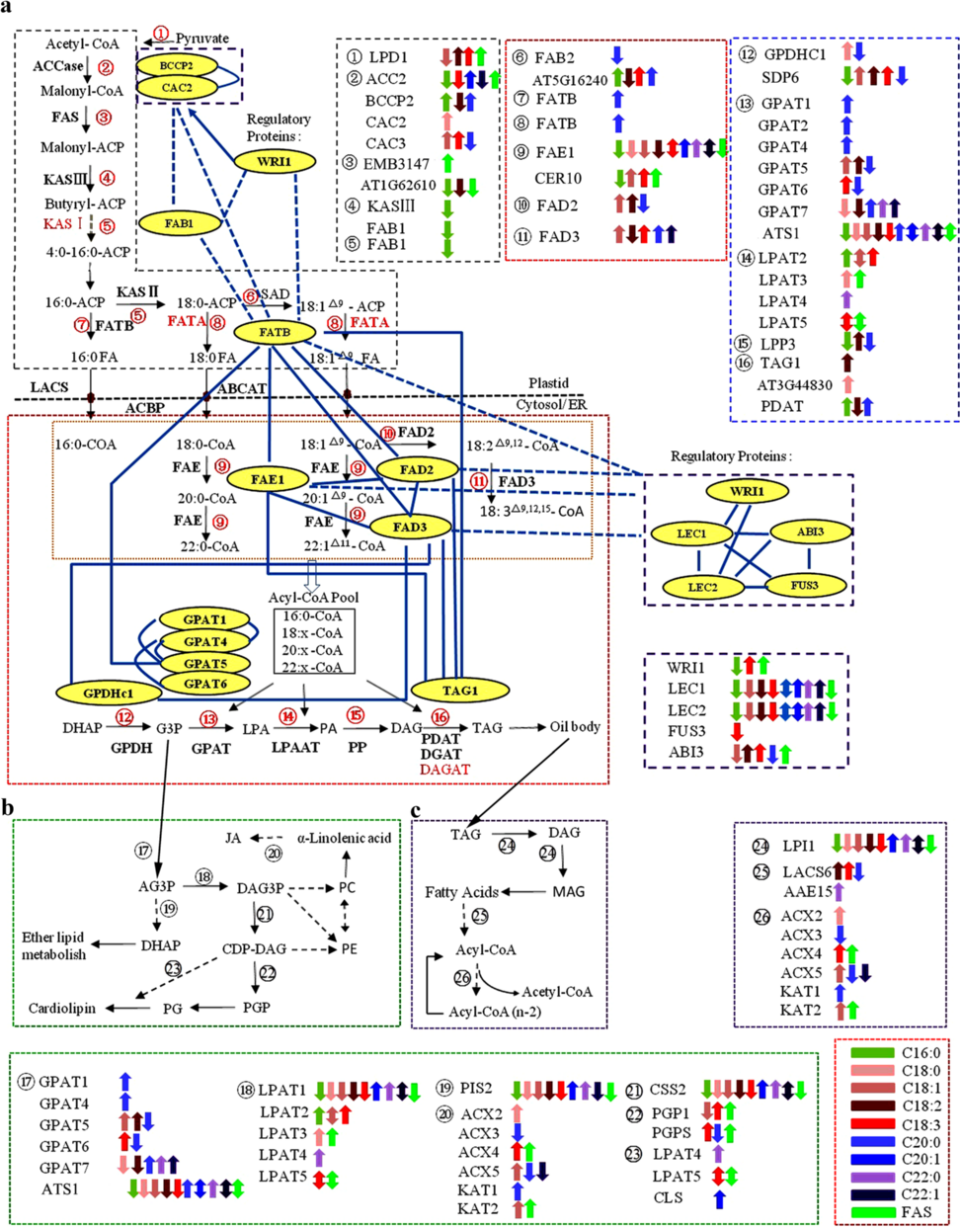
**Potential regulatory pathways and candidate genes associated with fatty acid synthesis in** ***B. napus*** **. a**, the fatty acid biosynthetic pathway; **b**, the phospholipid synthesis and other pathways; **c**, the triacylglycerol and fatty acid degradation. The black characters indicate genes detected in previous studies and also in this study, the red characters indicate genes not found in this study but in previous studies. The colored arrows indicate genes underlying a QTL which affects traits with additive effect, up-arrows indicate QTLs with positive additive and down-arrows a negative additive effect. The double-headed arrows indicate genes underlying different QTLs which affect the same trait with opposite additive effect at the same time based on the WinQTLcart2.5 and QTLNetwork_2.0 results (not combined results of the two). Different colors denote different traits as the bar shown at the lower right corner of the picture. The abbreviations of gene name are same with the following reference papers [30,37,38].

Among the 97 genes of *Arabidopsis* located in the CIs of 47 QTLs in *B. napus*, 57 were shown to have roles in 32 different pathways (Additional file 8). Among them, 31 genes were involved in at least one of the five pathways, including biosynthesis of unsaturated fatty acids, fatty acid metabolism, glycerophospholipid metabolism, fatty acid biosynthesis and alpha-linolenic acid metabolism, with 13, nine, nine, eight and six candidate genes, respectively (Additional file 9). The potential pathway for fatty acid synthesis in *B. napus*, which included 50 genes and the five regulatory factors mentioned above, was constructed by combining the main pathways that involved interaction of candidate genes as well as knowledge of fatty acid regulatory pathways in *Arabidopsis* (Figure 5). The candidate genes in the deduced pathways could be divided into three types: genes associated with the fatty acid biosynthetic pathway (Figure 5a), the phospholipid synthesis and other pathways (Figure 5b) and the triacylglycerol and fatty acid degradation (Figure 5c).

The diagrams of the fatty acid biosynthetic pathway in the plastid and the cytosol/endoplasmic reticulum could be divided into four categories: genes participating in early stages of fatty acid synthesis, fatty acid elongation and desaturation, triacylglycerol biosynthesis and regulators of fatty acid biosynthesis. At least 24 enzymes were involved in the plastidial fatty acid synthetic pathway [38]. *ACCase* was a major control point of the pathway among these enzymes [39,40], and four subunits of *ACCase* were detected (Figure 5a) and assigned to 13 QTLs: *ACC2* was assigned to seven QTLs: *qA3-3*, *qA5-2*, *qA8-5*, *qA8-6*, *qA9-4*, *qC3-1* and *qC9-3*; *BCCP2* to QTLs *qA3-1* and *qA8-6*; *CAC2* to QTL *qC9-4*; and *CAC3* to QTLs *qA4-7*, *qA8-1* and *qC6-1* (Additional file 4). Downstream from *ACCase*, fatty acids were constructed and elongated by each cycle with four reactions (steps 3–5). In step 3, two genes *EMB3147* and *AT1G62610* were found underlying the CIs of one (*qA5-3*) and four QTLs (*qA8-6*, *qA9-7*, *qC9-3* and *qC9-4*), respectively. Candidate genes of *KASIII* (*qA8-6*, *qA9-7*, *qC9-3* and *qC9-4*) and *FAB1* (*qA7-2*) in step 4 and *FAB1* in step 5 were also observed. This suggested that all these candidate genes in QTL CIs could affect the traits through their action in early stages of fatty acid synthesis.

Most C18:0-ACP produced by elongation was desaturated by the stearoyl-ACP desaturase in step 6, and *FAB2* (*qA8-1* and *qC6-1*) and *AT5G16240* (*qA3-1*, *qA8-6* and *qA10-2*) were found. Some important candidate genes were found to be associated with fatty acid elongation and desaturation; for example, *FAE1* and *CER10* appeared in step 9. *FAE1* was associated with QTLs *qA1-4* detected by C18:3 and *qA8-5* with all 10 traits, and *qC3-2* with nine traits but not C18:3. *CER10* was assigned to *qA4-3*, *qA5-2*, *qA10-1* and *qC9-6*, which were detected with C16:0, FAS, C18:3 and C18:1, respectively.

Triacylglycerol synthesis could be divided into four main steps [38,41]. DHAP could be reduced to G3P catalyzed by GPDH, and *GPDHc1* (*qA8-1*, *qC6-1* and *qC9-4*) and *SDP6* (*qA3-5*, *qA5-4*, *qC3-7* and *qC5-1*) were revealed in this step. From G3P to PA of steps 13 and 14, fatty acids were sequentially transferred from CoA to positions 1 and 2 of G3P catalyzed by *GPAT* and *LPAT*, respectively. *GPAT* was composed of 10 subunits [38], seven of which except for *GPAT3*, *GPAT8* and *GPAT9* were observed in this step. Among them, *GPAT1*, *GPAT2* and *GPAT4* were associated with QTL *qA8-2*; *GPAT5* with QTLs *qA3-5*, *qC3-7* and *qC5-1*; *GPAT6* with QTLs *qA4-7*, *qA8-1* and *qC6-1*; *GPAT7* with QTLs *qA8-6* and *qA10-3*; and *ATS1* with QTLs *qA3-3*, *qA5-2*, *qA8-5*, *qA8-6*, *qA9-4* and *qC9-3* (Additional file 4). *LPAT* was a main enzyme in step 14 with five subunits [38], and *LPAT2*, *LPAT3*, *LPAT4* and *LPAT5* were associated with four (*qA4-2*, *qA9-2*, *qA10-1* and *qC9-6*), two (*qA5-2* and *qC3-5*), one (*qA9-6*) and four (*qA3-6*, *qA5-3*, *qC3-8* and *qC5-1*) QTLs, respectively. *LPP3* was associated with QTLs *qA3-4* and *qC5-1* that catalyze dephosphorylation of PA to release DAG in step 15. Step 16 was the final step of TAG synthesis and *TAG1* (*qC8-1* and *qC9-3*), *AT3G44830* (*qC3-4*) and *PDA* (*qA3-1* and *qA8-6*) were observed in this step. Many genes or QTLs involved in one step indicated that the fatty acid synthesis in *B. napus* was very complicated.

Besides those genes that had direct activities in catalyzing fatty acid synthesis, some regulators (*LEC1*, *LEC2*, *FUS3*, *WRI1* and *ABI3*) underlying QTL CIs were also found (Figure 5). The interaction analysis indicated that *WRI1* directly affected *BCCP2* and *CAC2*, and indirectly affected *FAB1* and *FATB* in early stages of fatty acid synthesis regulation, while *WRI1*, *LEC1/2*, *FUS3* and *ABI3* all indirectly affected *FAE1*, *FAD2* and *FAD3*, which played important roles in later modifying and transporting processes. *LEC1* and *LEC2* were underlying the CIs of five QTLs (*qA3-3*, *qA8-4*, *qA8-6*, *qA9-6* and *qC9-3*) and six QTLs (*qA3-3*, *qA5-2*, *qA8-4*, *qA8-6*, *qA9-4* and *qC9-3*), respectively. Both *LEC1* and *LEC2* affected nine fatty acid compositions, except for C18:0, in different environments. *FUS3* located in the CI of the QTL *qC7-1* that was only associated with C18:3. *WRI1* located in the CIs of QTLs *qA4-3*, *qA5-2* and *qA10-1* was associated with C16:0, FAS and C18:3, respectively. *ABI3* was associated with three QTLs (*qA5-3*, *qC3-8* and *qC5-1*) and affected the levels of C18:3, FAS, C18:1, C18:2 and C20:0 (Figure 5). Moreover, the epistatic effects of major gene–gene interactions were also identified in fatty acid synthesis (Figure 5). For example, the major genes *FATB*, *FAE1*, *FAD2* and *FAD3* were all under the influence of each other and all could affect the gene *TAG1* in the final step of TAG synthesis, and then affected the level of different fatty acids (Figure 5). All these findings indicated that the regulatory pathway of fatty acid synthesis was a complex network, controlled by a large number of genes and also affected by gene–gene interactions.

Potential phospholipid synthesis (Figure 5b), and triacylglycerol and fatty acid degradation (Figure 5c), were also predicted in *B. napus* based on knowledge of acyl-lipid metabolism in *Arabidopsis* [31]. G3P was converted to DAG3P by sequential acylation reactions in steps 17 and 18, which was regulated by *GPAT* and *LPAT*, and the homologous genes involved in the two steps were very similar to the genes in steps 13 and 14, respectively. Then DAG3P was converted to PC or PE by a series of enzyme reactions. In the pathway of triacylglycerol and fatty acid degradation, a number of homologous genes were also found, and the six genes in step 26 were the same as the genes in step 20. Moreover, gene *LPI1*, which functioned in degradation of TAG to MAG, affected the level of nine traits but not C20:0.

In addition to the 47 QTLs with candidate genes mapped in the CIs, the remaining 25 QTLs also affected the concentrations of one or two fatty acids. QTL *qA4-4* on chromosome A4 with additive effect on traits C16:0 and C18:0 might be assigned to step 5 (Figure 5) with potential ketoacyl ACP synthetase activity. QTL *qC5-2* might be assigned to step 11 with a similar function to *FAD3*, as the QTL had a consistent additive effect on traits C18:2 and C18:3. QTL *qC9-5* might be a pleiotropic locus with a similar function to *FAE1* as it had an additive effect on traits C18:0 and C22:1. The remaining 22 QTLs only had additive effects on one trait. Based on the premise that plastidic synthesis and modification should result in consistent QTL effects on products in any of the individual sets of saturated fatty acids [42], *qA7-3*, *qA7-4* and *qC9-1* affected *C16:0*, and *qA1-5* and *qA6-2* affected C18:0, which allows us to assign these QTLs tentatively to early stages of synthesis. The other 17 QTLs may be assigned to desaturation and elongase steps, as they affected C18:3, C20:0, C20:1, C22:0, C22:1 and FAS. Generally, it is important to take into account all the preceding synthetic and modification steps where a QTL could be identified for a particular fatty acid [42]; however, assigning a specific enzyme activity to these QTLs is relatively complex.

## Discussion

A total of 72 QTLs and a large number of pairs of epistatic loci for 10 fatty acid compositions were identified using QTL analysis. Then 234 homologous genes were mapped in the CIs of 47 QTLs. QTL mapping and interaction analysis of candidate genes enabled the construction of a regulatory pathway that controlled the different concentrations of fatty acids in *B. napus*.

Single QTL mapping of each fatty acid concentration showed that QTLs on A8 and C3 were highly correlated. QTL *qA8-5* on A8 affected concentrations of all 10 types of fatty acids, and explained PV of individual fatty acids ranging from 15.89% for C20:1 to 47.55% for C20:0, and QTL *qA8-6* affected seven fatty acids; meanwhile, both QTLs *qC3-2* and *qC3-3* on C3 affected nine fatty acids but not C18:3. All these results revealed that these QTLs were major QTLs, consistent with previous studies [6,9,10]. For the two QTLs on C3, the alleles from ‘Ningyou 7’ conferred higher levels of C20:0, C20:1, C22:0 and C22:1, but had negative effects on levels of C16:0, C18:0, C18:1 and C18:2. The group C16:0, C18:0, C18:1 and C18:2 were positively correlated with each other, and likewise for the group C20:0, C20:1, C22:0 and C22:1; however, the two groups showed a negative correlation (Table 2). The fact that QTLs clustered in certain linkage groups and controlled different traits with opposite effects, corroborated strongly with the significant positive and negative correlations among the fatty acids analyzed. Besides the two major QTLs on A8 and C3, another 15 QTLs also affected more than one fatty acid. Moreover, the number of QTLs associated with a single fatty acid in the present study ranged from nine (C18:2, C20:0 and C22:1) to 21 (C18:3), and a total of 72 QTLs for all 10 traits detected were more than that from previous studies [6-10], with a number of new QTLs for different fatty acids. No QTLs for C16:0 were found on A4, A7 and C9 previously [6,8,10], suggesting that the 10 QTLs on these chromosomes in the TN population were all new QTLs. The unsaturated fatty acids were synthesized as a result of the fatty acid desaturation pathway, starting from C18:0; however, only a few QTLs for C18:0 were detected in previous studies. Zhao *et al*. identified two QTLs for C18:0 on A6 and A7 [8], Smooker *et al*. identified one QTL on A9 [9], and Burns *et al.* detected four QTLs respectively on A1, A6, A7 and A8 [6]. Herein, 12 QTLs across seven chromosomes were detected, and the QTLs on A4 (one QTL), A10 (two QTLs), C3 (four QTLs) and C9 (two QTLs) were new. QTLs for C18:1 were identified on 14 chromosomes except A4, A6, C2, C5 and C7 [6-10]. In the present study, there were 12 QTLs associated with C18:1 across seven chromosomes, nine of which also affected other fatty acids. QTLs for C18:2 were identified on 15 chromosomes except A4, A10, C5 and C9 [6,8-10], indicating that the two QTLs (*qC5-1* and *qC5-2*) for C18:2 on C5 were new. There were 21 QTLs for C18:3 identified here, and three QTLs (*qA2-1*, *qA2-2* and *qA2-3*) mapped on A2 and one QTL (*qC7-1*) on C7 were new QTLs, but the former 18 QTLs were distributed across 14 chromosomes excluding A2, A3, A9, C6 and C7 [6-10]. Many QTLs for C22:1 were identified on 14 chromosomes excluding A4, A5, A7, C1 and C5 [4-6,8-10,43]. Two major QTLs associated with C22:1 on A8 and C3 chromosomes were observed in the present study, which was consistent with previous studies [5,9], and two new QTLs (*qA4-1* and *qA7-1*) were mapped on A4 and A7 chromosomes (Table 3).

Generally, additive effects are considered as the main factors contributing to variations in quantitative traits [44,45]. However, epistatic effects can also play an important role in complex traits in *B. napus* [46,47]. Epistatic QTLs was found to explain PV ranging from 0.01% for C18:2 to 25.47% for C20:1 (Additional file 6), showing similar trends to previous studies [48]. Moreover, interactions among 395 loci were identified by GMM, including 34 pairs of digenic and 361 pairs of trigenic interactions (Additional file 7). A number of candidate genes were mapped to the CIs of these QTLs. These results showed that epistasis may substantially contribute to variation in oil concentration in different crops.

The regulatory pathway constructed in this study was based on QTL information and the interaction of candidate genes associated with the single and epistatic QTLs of 10 fatty acids, as well as previous knowledge concerning *Brassica* species and *Arabidopsis* [31,42]. A large number of important genes were found to be candidate genes for the processes of fatty acid metabolism. Among the 50 genes and five regulatory factors detected, 31 genes (62.0%) affected more than one trait, and *FAE1* and *ATS1* affected nine (but not C20:0) and 10 traits, respectively. Previous studies found that *FAE1* affected oil composition in several steps of seed fatty acid synthesis and modification pathways in *B. oleracea* [42] and *B. napus* [30,49]. In *B. napus*, the orthologs of *FAD2* and *FAD3* were mapped in different linkage groups of the genome. *BnaFAD2* loci were mapped in linkage groups A1, A5, C1 and C5 [28], and *BnaFAD2* located in different linkage groups was found to be responsible for the high C18:1 level [29] and erucic acid content [9]. It seemed clear that a major functional variant of *FAD2* orthologs in the A genome was located on linkage group A5, but unclear whether *FAD2* loci were present in the *Brassica* C genome [8,29]. The ortholog of *FAD3* was identified in the control of C18:3 level in *B. napus*, and a specific marker of *fad3* was located in the CI of the QTL for C18:3 in linkage group A1 [50]*.* In the present study, *FAD2* was located in three QTLs CIs: *qA3-5* by C16:0 and C18:1, *qC3-7* by C20:0, and *qC5-1* by C18:2 and C20:0 using *in silico* mapping. The homologous genes of *FAD3* were mapped in the CIs of seven QTLs: *qA3-5* by C16:0 and C18:1; *qA5-3* by C18:3 and FAS; *qA5-4* by C18:1 and C18:3; *qA8-6* by C18:1, C18:2, C20:0, C20:1, C22:0, C22:1 and FAS; *qA10-3* by C18:0; *qC3-7* by C20:0; and *qC5-1* by C18:2 and C20:0. Taken together, these findings increased the understanding of the important genes affecting the processes of fatty acid metabolism in *B. napus*.

Despite the multiple studies on the function of several important genes in fatty acid biosynthesis [23-26], the regulatory mechanism of the pathway is still not well understood in *B. napus*. Five regulatory factors (*LEC1*, *LEC2*, *WRI1*, *FUS3* and *ABI3*) were involved in the process concerning the fatty acid biosynthetic pathways, which might correspond to new important findings in this study. Both *LEC1* and *LEC2* could act as positive regulators upstream of *ABI3* and *FUS3* in the control of seed maturation [51-53], and several studies revealed that these four regulatory factors might have a function in regulation of fatty acid metabolism [37,51-54]. *WRI1*, which acted downstream of *LEC2*, controlled expression of a subset of genes involved in fatty acid biosynthesis [33], and overexpression of *WRI1* caused an increased TAG level in both seeds and leaves of *Arabidopsis* [34]. In this study, each of the five regulatory factors was under the influence of at least two other regulatory factors, and *LEC1* and *LEC2* were especially affected by all four other regulatory factors (Figures 4 and 5). Moreover, all five regulatory factors were associated with C18:3, but none affected C18:0. Conversion of acetyl-CoA to malonyl-CoA by acetyl carboxylase (ACCase) is one of the most committed steps in fatty acid biosynthesis [39,40], and reduction of ACCase activity caused a decrease of fatty acid content in the range of 1.5–16.0% in transgenic seeds of oilseed crops [55]. Most of the enzymes involved in *de novo* fatty acid synthesis seemed to be affected by *WRI1* [33], and only *WRI1* of the five regulatory factors had a direct impact on transcription levels of genes in the plastidial fatty acid synthetic pathway [38]. As a result of the interaction analysis of candidate genes, two genes *CAC2* and *BCCP2*, which encoded subunits of ACCase, were found to be under the influence of *WRI1*. In the process of fatty acid elongation and desaturation, no candidate genes were shown to be under the influence of the five regulatory factors in this study (Figure 5). Among the targets of the five regulatory factors, *LFY* might represent a key factor mediating the regulatory action of elongation and desaturation processes, as it was directly affected by all five regulatory factors and also regulated important downstream genes, including *FAD2*, *FAE1* and *FATB*, and these three genes regulated a series of downstream genes (Figure 4). *LFY* was arguably the most important floral meristem identity gene, which encoded a plant-specific transcription factor and controlled multiple aspects of floral morphogenesis [56,57]. It was striking that *LFY* played an important role in the regulatory pathway of fatty acid metabolism, and is worthy of future study.

Overall, these results provided important new insights into the regulatory model for the control of oil synthesis in *B. napus* and enhanced our understanding of the fatty acid synthesis pathways.

## Conclusions

In this study, 72 individual QTLs and a large number pairs of epistatic interactions contributing to fatty acid biosynthesis were identified. By using *in silico* mapping analysis, 234 homologous genes of *A. thaliana* that could be involved in fatty acid metabolism within the CIs of 47 QTLs were found. After QTL mapping and candidate gene analysis, a potential regulatory network controlling fatty acid metabolism was revealed in *B. napus*. Our results provided new insights into the regulatory model for the control of oil synthesis in *B. napus*.

## Methods

### Plant materials and field experiments

The segregating DH population (the TN DH population), with 202 DH lines derived from the cross ‘Tapidor’ × ‘Ningyou7’ was constructed by Qiu *et al*. [5]. The DH lines as well as their parents were grown in six independent environments in China. The population was grown at Wuhan (W1) for one year (September 2007 to May 2008, 07 W1), Huanggang (W2) for two years (September 2008 to May 2009, 08 W2; September 2009 to May 2010, 09 W2) and Qichun (W3) for one year (September 2009 to May 2010, 09 W3) in Hubei Province; and Dali for two years (September 2007 to May 2008, 07D; September 2009 to May 2010, 09D) in Shaanxi Province. Wuhan, Qichun and Huanggang were the experiment bases of Huazhong University of Science and Technology, and Dali was the experiment base of Hybrid Rapeseed Research Center of Shaanxi Province. No specific permissions were required for the field trials. For each field trial, all lines were planted in a randomized complete-block designed with three replicates, and each plot contained 30 plants per genotype [58].

### Measurements of fatty acid composition

Bulked seed samples (200 mg) from each of replicates in the three environments (07D, 07 W1 and 08 W2) were analyzed for their fatty acid compositions by gas liquid chromatography according to Rücker and Röbbelen [59]. In three other environments (09D, 09 W2 and 09 W3), fatty acid compositions were determined by near-infrared reflectance spectroscopy (NIRS) using standard methods [60] and all saturated fatty acid compositions in these three environments were considered as one trait named FAS.

### Linkage map construction and map alignment with *Arabidopsis*, *B. rapa* and *B. oleracea*

The TN genetic linkage map of *B. napus*, which was introduced by Jiang *et al*. was updated in the present study [61]. A total of 932 markers were mapped to the new linkage map generated with the TN DH population using JoinMap_4.0 [62]. Detailed information of TN genetic linkage map was summarized in Additional file 3. Of the 932 linked markers, 429 with known sequence information or corresponding to *Arabidopsis* genes were used as anchored markers to carry out map alignment between *B. napus* and *Arabidopsis* according to the method of Long *et al*. [21]. If more than three homologous sequence-informative markers in the TN DH population were closely linked within one conserved block of *Arabidopsis* as described by Schranz *et al*. [17], a synteny block was considered to exist. If there were only one or two sequence-informative marker(s), it was recognized as an insertion segment. The homologous genes underlying the block or island were considered to be candidate genes if they were just underlying the QTL CI. There were 111 key genes (824 homologous genes) (Additional file 4) involved in fatty acid metabolism of *Arabidopsis* collected from the TAIR website [63] and *in silico* mapping in the genetic map as described by Long *et al*. [21]. The comparative mapping analysis between *B. napus* and *B. rapa/B. oleracea* was done using BLAST analysis on the *Brassica* Database [64] or *B. oleracea* Database [65]. The sequence of markers in the TN DH linkage map was used as a query sequence. All available sequence-informative markers on *B. napus* were subjected to BLASTn analysis to identify their physical positions on *Arabidopsis*, *B. rapa* and *B. oleracea* chromosomes*.* Then the Brassicaceae building blocks that corresponded to each informative marker were identified. The candidate gene(s) in *B. napus*, *B. rapa* and *B. oleracea* for each informative marker were also identified.

### QTL and epistasis analysis

QTL analysis was done using the QTLNetwork_2.0 software described by Yang *et al*. [66,67] and WinQTLCart_2.5 [68], and epistasis QTL mapping analyses were performed using the QTLNetwork_2.0 and GMM [69]. For QTLNetwork_2.0, a mixed linear model was used to identify QTLs at 2-cM intervals with a window size of 10 cM. Two-dimensional (2D) genome scans were used to search for multiple interacting QTLs. For each trait, a genome-wide threshold value of the F-statistic (*P* = 0.05) for declaring the presence of a QTL was estimated by 1000 random permutations [70]. A Bayesian method with Gibbs sampling was used to estimate QTL effects [71]. The sum of individual PV explained by each QTL was calculated as the total PV explained by all QTLs for each trait. Based on genetic effects, mapped epistatic QTLs comprised three types: interactions between two QTLs with additive effects (AA), interactions between a QTL with additive effect and a locus without significant additive effect (AN or NA), and interactions between two loci within non-QTL (NN) [45]. For WinQTLCart_2.5, composite interval mapping (CIM) was used with a significance threshold of *P* = 0.05 using the permutation test method [68], based on 1000 runs of randomly shuffling the trait values [72]. QTL CIs were determined by 2-LOD intervals surrounding the QTL peak. QTLs for different traits that mapped to the same region with overlapping CIs were assumed to be the same [58,73]. For GMM, two and three loci interactions were detected with default parameters. For the QTL comparison between different populations, QTLs detected in this study were preliminarily compared with those in other publications, because few common markers were available between TN and other populations. If one or more QTLs were identified on a chromosome in this study but not in previous studies, these were considered as new QTLs.

### The genetic interaction analysis candidate genes

The network was constructed and analyzed using Cytoscape_V2.6.3 with the Agilent Literature Search Plug-in [74]. The degree of connectivity, the clustering coefficient, the network density and the diameter were designed according to Toubiana *et al.* [75]. Search controls option by using the default parameter settings and extraction controls option with appropriate choice and choosing *Arabidopsis* in concept lexicon, the network was laid out by using group attributes layout.

### Construction of potential controlling pathways of fatty acids in *B. napus*

To identify candidate genes underlying the QTL CIs involved in the fatty acid regulatory pathways, the Kyoto Encyclopedia of Genes and Genomes (KEGG) database was applied to identify pathways in which these genes were involved [76,77]. Potential controlling pathways of fatty acids in *B. napus* were inferred based on the pathways of fatty acid synthesis within *Arabidopsis* [31,38] and other plants [42,78] and also using gene interaction analysis and QTL epistasis analysis.

## Additional files

Additional file 1:
**Single QTL detection by WinQTLCart_2.5 in TN DH population.**
Additional file 2:
**Single QTL detection by QTLNetwork_2.0 in TN DH population.**
Additional file 3:
**The TN DH genetic linkage map and its syntenic segmental alignment with the** ***Arabidopsis*** **genome.**
Additional file 4:
**Positions of homologous genes of** ***Arabidopsis*** **mapped on TN DH linkage map by** ***in silico*** **mapping and orthologous genes between** ***B. napus*** **and** ***B. rapa/B. oleracea*** **.**
Additional file 5:
**Demonstration of a complex epistatic network for fatty acids in** ***B. napus*** **.**
Additional file 6:
**Epistatic QTLs for fatty acid compositions detected by QTLNetwork_2.0 in TN DH population.**
Additional file 7:
**Digenic and trigenic interactions for fatty acid compositions detected by genotype matrix mapping software ver2.1 in TN DH population.**
Additional file 8:
**The distribution of 57 candidate genes in 32 different pathways by the KEGG analysis.**
Additional file 9:
**Five KEGG pathway maps of candidate genes in** ***Arabidopsis.***

## Abbreviations

*ABCAT*: ABC acyl transporter
*ACBP*: Acyl CoA binding protein
*ACCase*: Acetyl-CoA carboxylase
CI: Confidence interval
DAG: Diacylglycerol
*DAGAT*: Diacylglycerol transacylase
*DGAT*: Acyl-CoA,diacylglycerol acyltransferase
DH: Doubled haploid
*FAE*: Fatty acid elongase
FAS: Fatty acid synthase
*FAT*: Fatty ACP thioesterase
G3P: Glycerol-3-phosphate
GMM: Genotype Matrix Mapping ver2.1software
*GPAT*: Glycerol-3-phosphate acyltransferase
*GPDH*: NAD-dependent glycerol-3-phosphate dehydrogenase
JA: Jasmonate
*KAS*: Ketoacyl-ACP synthase
*LACS*: Long-chain acyl-CoA synthetase
LPA (AG3P): Lysophosphatidic acid
*LPAAT*: 1-acylglycerol-3-phosphate acyltransferase
MAG: Monoacylglycerol
PA (DAG3P): Phasphatidic acid
PC: Phosphatidylcholine
*PDAT*: Phospholipid,diacylglycerol acyltransferase
PE: Phosphatidylethanolamine
PG: Phosphatidylglycerol
PGP: Phosphatidyglycerophosphate
*PP*: Phosphatidate phosphatase
PV: Phenotypic variance
QTL: Quantitative trait loci
*SAD*: Stearoyl-ACP desaturase
TAG: Triacylglycerol
