# Supplementary material for: New insights into the genetic networks affecting seed fatty acid concentrations in Brassica napus
Source: BMC Plant Biol. 2015 Mar 27;15:91. doi: 10.1186/s12870-015-0475-8 (PMC4377205; doi:10.1186/s12870-015-0475-8)
Supplement: Additional file 5: — Demonstration of a complex epistatic network for fatty acids in B. napus . [file 12870_2015_475_MOESM5_ESM.docx]

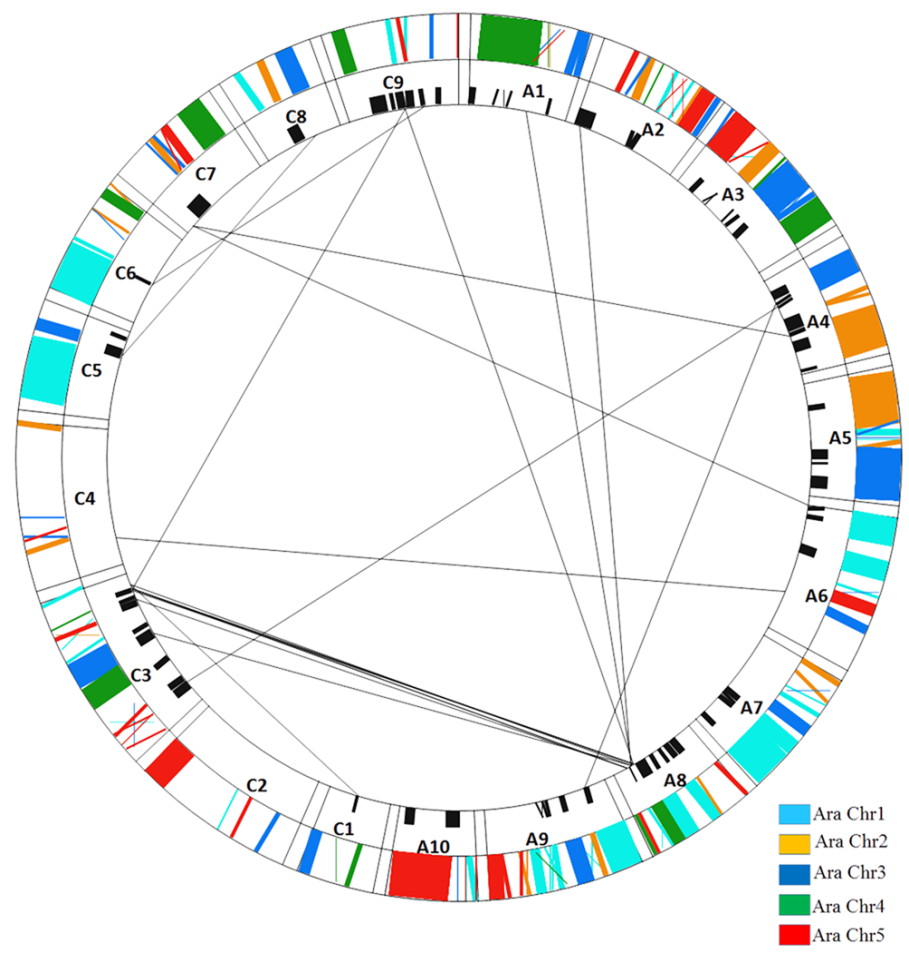


**Additional file 5:** **Demonstration of a complex epistatic network for fatty acids in *B. napus*.** The 19 sectors of the disc indicate the 19 linkage groups of the TN DH linkage map. The boxes of different colors at the outer edge represent the different pseudo-chromosome fragments of *Arabidopsis* that are aligned with chromosome fragments of *B. napus.* The black lines perpendicular to the linkage group bars at the inner edge indicate the confidence intervals of QTLs for fatty acids. The long, thick, black lines indicate epistatic interactions for fatty acids.
