# Supplementary material for: New insights into the genetic networks affecting seed fatty acid concentrations in Brassica napus
Source: BMC Plant Biol. 2015 Mar 27;15:91. doi: 10.1186/s12870-015-0475-8 (PMC4377205; doi:10.1186/s12870-015-0475-8)
Supplement: Additional file 6: — Epistatic QTLs for fatty acid compositions detected by QTLNetwork_2.0 in TN DH population. [file 12870_2015_475_MOESM6_ESM.docx]

**Additional file 6: Epistatic QTL for fatty acid compositions in TN DH population**

| Trait | QTL_i | interval_i | position_i | range_i | QTL_j | interval_j | position_j | range_j | AA | PV(aa,%) |
| --- | --- | --- | --- | --- | --- | --- | --- | --- | --- | --- |
| 16:0 | **4-10** | **IGF3365B-SN13034** (***qA4-3***) | 26.5 | 25.7-28.5 | **9-11** | **PW123BE-HBR178**  (***qA9-1***) | 23.4 | 18.3-25.3 | 0.0449 | 4.99 |
|  | **8-23** | **HG-FAE1-A8-SS1702** (***qA8-5***) | 70.7 | 70.1-71.7 | **13-59** | **HG-FAE1-C3-OL13H09** (***qC3-3***) | 136.9 | 136.5-137.9 | 0.0790 | 3.18 |
|  | **8-23** | **HG-FAE1-A8-SS1702** (***qA8-5***) | 70.7 | 70.1-71.7 | **19-15** | **HBR144-EM17ME21-400** (***qC9-3***) | 60.1 | 58.8-61.1 | 0.0515 | 3.39 |
|  | **13-59** | **HG-FAE1-C3-OL13H09** (***qC3-3***) | 136.9 | 136.5-137.9 | **19-15** | **HBR144-EM17ME21-400** (***qC9-3***) | 60.1 | 58.8-61.1 | 0.0359 | 0.66 |
| subtotal |  |  |  |  |  |  |  |  |  | 12.22 |
| 18:0 | **4-15** | **BRMS-276-HR-C001-A4** (***qA4-4***) | 30.4 | 29.5-32.3 | **13-9** | **HBR032-BRMS-106**  (***qC3-4***) | 24.5 | 16.0-26.0 | -0.0427 | 0.76 |
|  | **8-23** | **HG-FAE1-A8-SS1702** (***qA8-5***) | 70.7 | 69.4-71.7 | **13-59** | **HG-FAE1-C3-OL13H09** (***qC3-3***) | 136.9 | 135.5-138.9 | 0.0763 | 3.05 |
| subtotal |  |  |  |  |  |  |  |  |  | 3.76 |
| 18:1 | **8-23** | **HG-FAE1-A8-SS1702** (***qA8-5***) | 70.7 | 70.1-71.7 | **13-59** | **HG-FAE1-C3-OL13H09** (***qC3-3***) | 136.9 | 135.5-138.9 | 4.0999 | 11.59 |
|  | **8-31** | **HR-S4-295-HR-C002-A8** (***qA8-6***) | 78.3 | 77.4-80.3 | **13-59** | **HG-FAE1-C3-OL13H09** (***qC3-3***) | 136.9 | 135.5-138.9 | 2.1270 | 10.70 |
| subtotal |  |  |  |  |  |  |  |  |  | 22.29 |
| 18:2 | 5-24 | BRAS095-CNU398 | 58.6 | 58.3-58.9 | **5-37** | **KBRH001C24-16-HG4-WG2E2** (***qA5-3***) | 73.3 | 71.4-73.3 | -1.1373 | 1.02 |
|  | 5-24 | BRAS095-CNU398 | 58.6 | 58.3-58.9 | 5-24 | BRAS095-CNU398 | 58.6 | 58.3-58.9 | 1.1296 | 0.01 |
| subtotal |  |  |  |  |  |  |  |  |  | 1.03 |
| 18:3 | **2-2** | **CB10355-BRMS-228** (***qA2-1***) | 12.3 | 6.3-15.8 | **8-27** | **SR7178-HBR016**  (***qA8-5***) | 74.2 | 73.1-75.0 | 0.0142 | 0.07 |
|  | **4-27** | **BRMS-105-BRMS-054** (***qA4-6***) | 63.0 | 60.5-65.2 | **17-4** | **SNRH63-CNU400**  (***qC7-1***) | 30.8 | 18.8-37.2 | -0.0906 | 0.80 |
|  | **6-1** | **OL11F12A-ZAAS92A** (***qA6-1***) | 2.0 | 0.0-4.0 | **17-4** | **SNRH63-CNU400**  (***qC7-1***) | 30.8 | 18.8-37.2 | -0.0132 | 0.03 |
|  | **15-11** | **IGF3112A-RA2F11** (***qC5-1***) | 68.0 | 63.7-72.0 | 18-22 | IGF3369A-EM18ME6-100 | 61.1 | 54.5-65.3 | -0.1320 | 1.84 |
| subtotal |  |  |  |  |  |  |  |  |  | 2.71 |
| 20:1 | **8-25** | **HBR095-HBR074**  (***qA8-5***) | 73.1 | 71.7-73.7 | **13-33** | **PW143-JICB2040_200**  (***qC3-7***) | 89.5 | 85.5-91.5 | -0.6828 | 3.83 |
|  | **8-25** | **HBR095-HBR074**  (***qA8-5***) | 73.1 | 71.7-73.7 | **13-61** | **HS-AU8-BRMS-093**  (***qC3-3***) | 142.3 | 140.4-144.3 | -2.9752 | 25.47 |
|  | **11-19** | **EM18ME23-300-CB10258** (***qC1-1***) | 34.3 | 33.0-35.9 | **13-61** | **HS-AU8-BRMS-093**  (***qC3-3***) | 142.3 | 140.4-144.3 | -0.4669 | 1.57 |
|  | 1-37 | OL12F11-ZNS06M30-150 | 60.3 | 59.7-61.6 | **8-25** | **HBR095-HBR074**  (***qA8-5***) | 73.1 | 71.7-73.7 | 0.5061 | 3.44 |
|  | 6-58 | CNU219-IGF3239D | 92.7 | 90.1-98.4 | 14-8 | EM06ME07-230-SS2277 | 28.3 | 25.9-32.1 | -0.7069 | 3.95 |
| subtotal |  |  |  |  |  |  |  |  |  | 38.26 |
| 22:0 | **8-23** | **HG-FAE1-A8-SS1702** (***qA8-5***) | 70.7 | 69.4-71.7 | **13-58** | **JICB0633-HG-FAE1-C3** (***qC3-3***) | 135.5 | 133.8-136.9 | 0.0385 | 4.37 |
| subtotal |  |  |  |  |  |  |  |  |  | 4.37 |
| 22:1 | **16-14** | **SR12387-EM14ME28-200** (***qC6-3***) | 44.0 | 41.2-45.3 | **19-26** | **SNRG42-HR-C017-C9**  (***qC9-5***) | 80.8 | 79.7-82.8 | -0.9803 | 0.14 |
|  | **8-31** | **HR-S4-295-HR-C002-A8** (***qA8-6***) | 78.3 | 77.4-80.3 | **13-53** | **HBR014-MR049**  (***qC3-2***) | 128.1 | 126.1-129.7 | -0.3275 | 1.98 |
|  | **8-31** | **HR-S4-295-HR-C002-A8** (***qA8-6***) | 78.3 | 77.4-80.3 | **13-59** | **HG-FAE1-C3-OL13H09** (***qC3-3***) | 136.9 | 135.5-138.9 | -2.7229 | 2.87 |
| subtotal |  |  |  |  |  |  |  |  |  | 4.99 |
| FAS | **8-23** | **HG-FAE1-A8-SS1702**  (***qA8-5***) | 70.7 | 70.1-71.7 | **13-59** | **HG-FAE1-C3-OL13H09** (***qC3-3***) | 136.9 | 133.8-136.9 | 0.0609 | 1.86 |
| subtotal |  |  |  |  |  |  |  |  |  | 1.86 |

QTL_i and QTL_j – The two QTL involved in epistatic interaction. QTL is named with chromosome (1-19) on which the QTL relevant following the marker intervals. For example, QTL 4-10 means it locates at the 10th marker interval of the A4 chromosome.

Markers with bold indicated that the locus was associated with the QTL with additive effect, and the QTL was showed under the locus.

The epistatic interactions with positive indicated that the combination of alleles at interacting loci from the same parent, while the epistatic interactions with negative effect indicated that the combination of alleles at interacting loci from different parents.
