# Supplementary material for: New insights into the genetic networks affecting seed fatty acid concentrations in Brassica napus
Source: BMC Plant Biol. 2015 Mar 27;15:91. doi: 10.1186/s12870-015-0475-8 (PMC4377205; doi:10.1186/s12870-015-0475-8)
Supplement: Additional file 8: — The distribution of 57 candidate genes in 32 different pathways by the KEGG analysis. [file 12870_2015_475_MOESM8_ESM.docx]

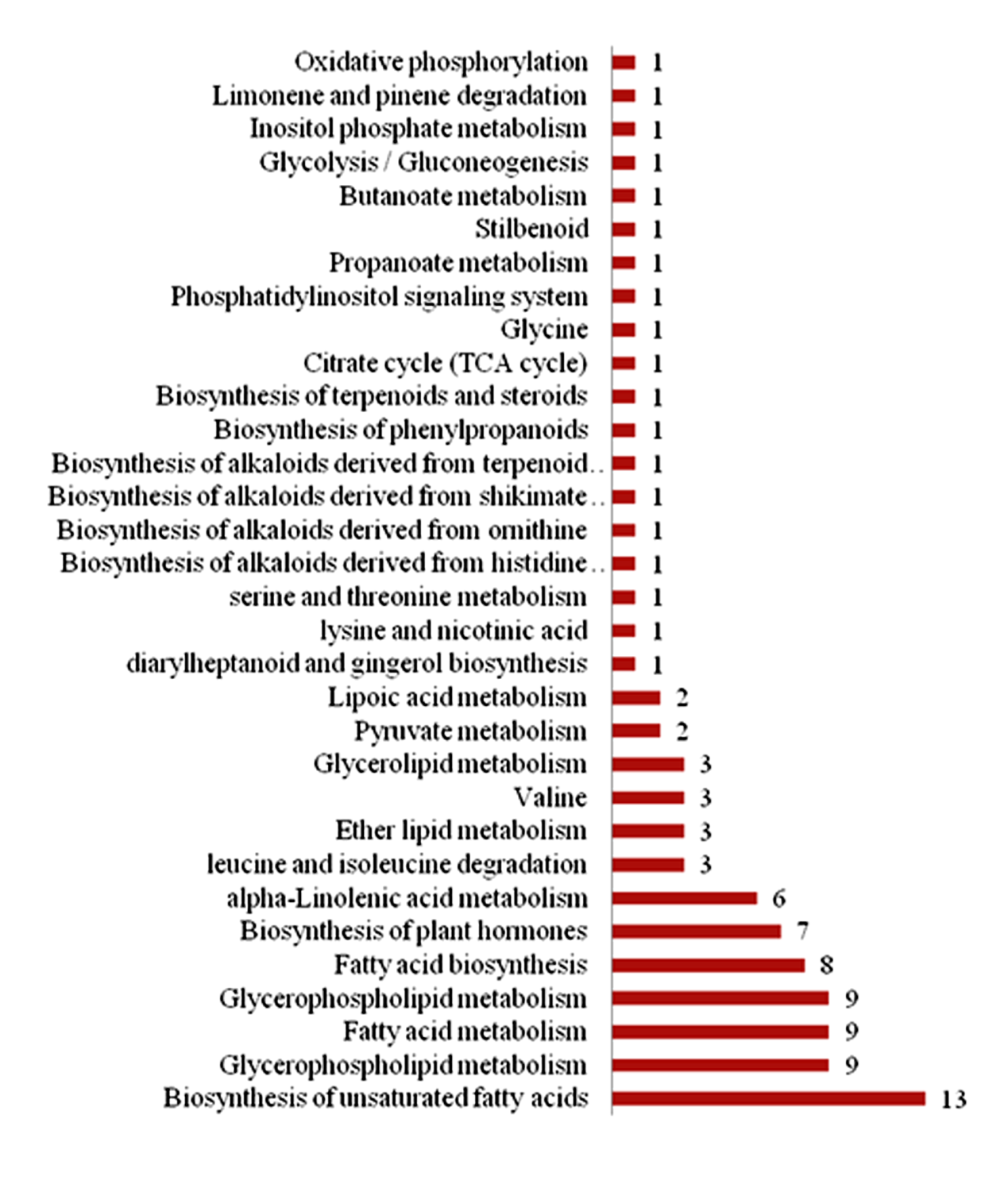


**Additional file 8**: The distribution of 57 candidate genes in 32 different pathways by the KEGG analysis. The x-axis shows the number of genes which have roles in different pathways, and the y-axis shows different pathways.
