# Supplementary material for: New insights into the genetic networks affecting seed fatty acid concentrations in Brassica napus
Source: BMC Plant Biol. 2015 Mar 27;15:91. doi: 10.1186/s12870-015-0475-8 (PMC4377205; doi:10.1186/s12870-015-0475-8)
Supplement: Additional file 9: — Five KEGG pathway maps of candidate genes in Arabidopsis. [file 12870_2015_475_MOESM9_ESM.docx]

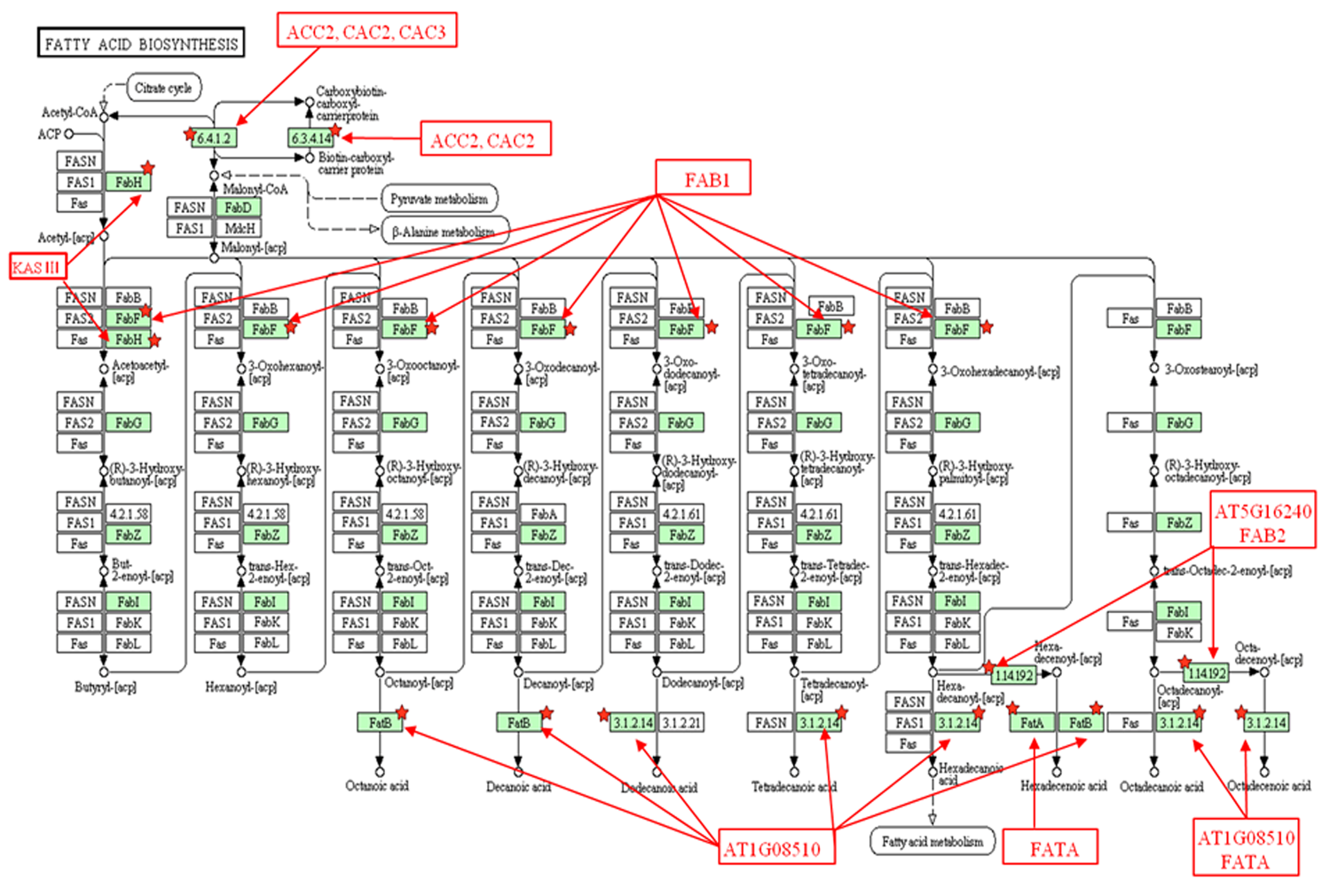


KEGG pathway of fatty acid biosynthesis in *Arabidopsis*.


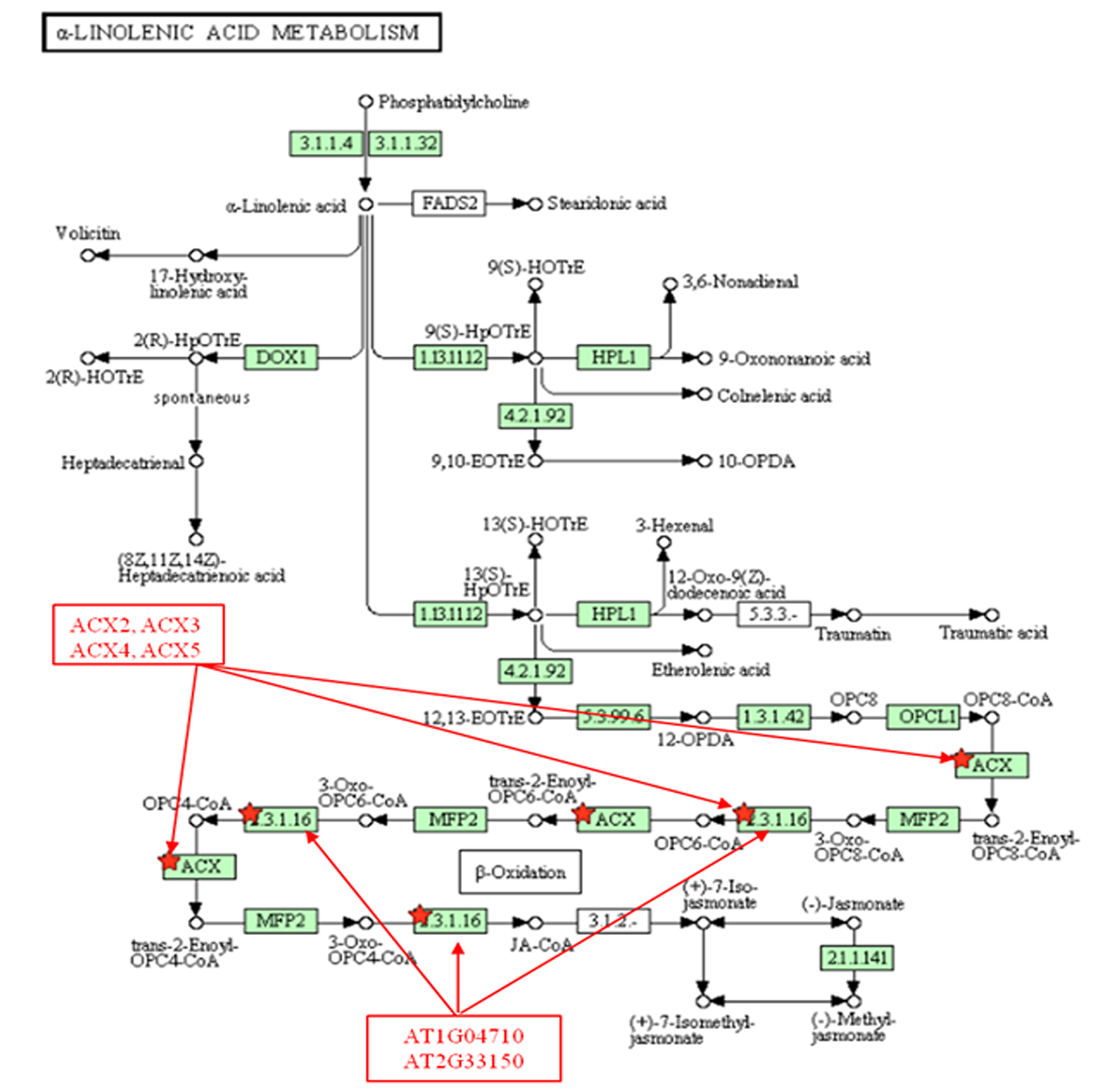


KEGG pathway map of alpha-linolenic acid metabolism


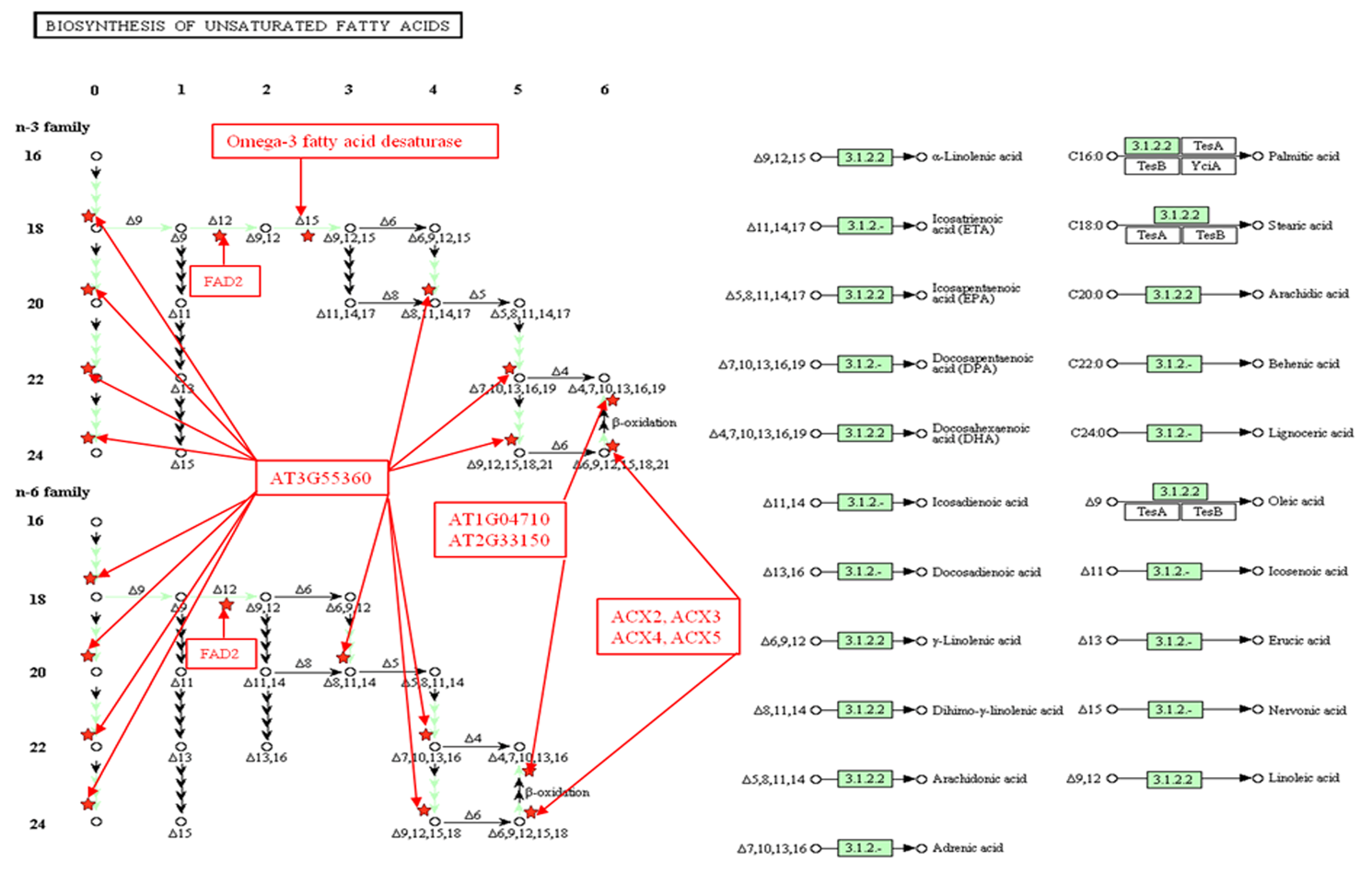


KEGG pathway of biosynthesis of unsaturated fatty acids


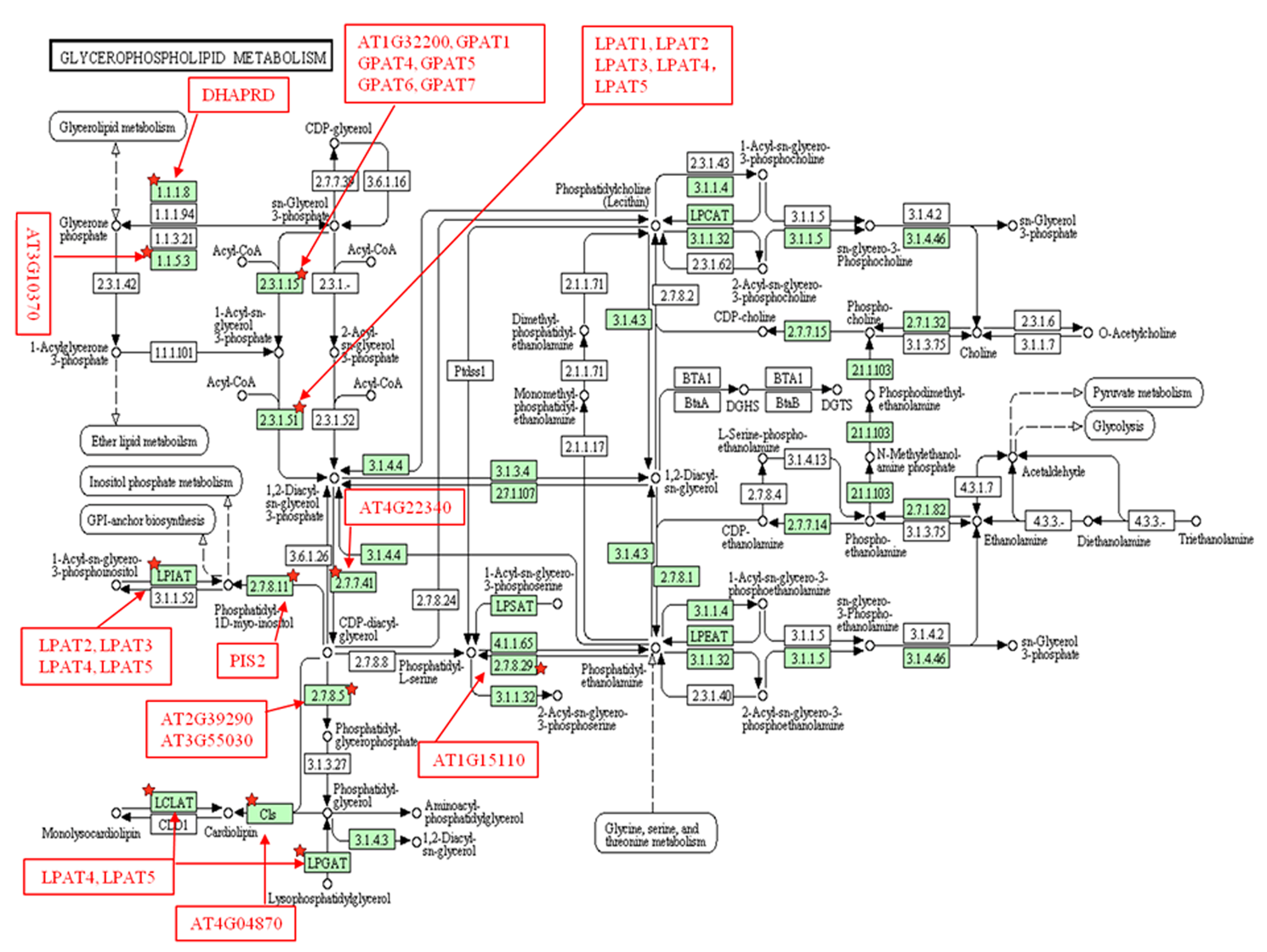


KEGG pathway of glycerophospholipid metabolism


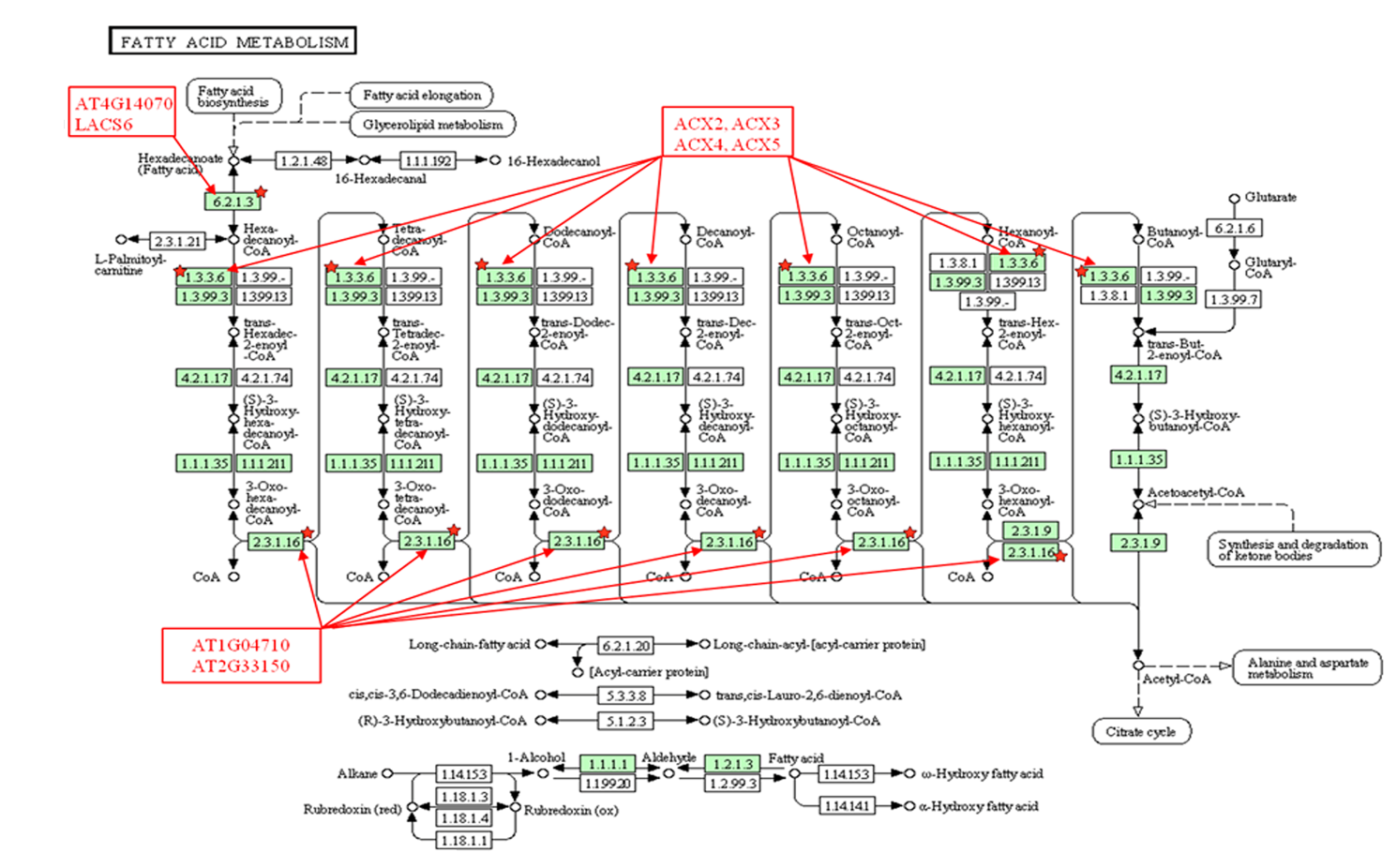


KEGG pathway of fatty acid metabolism

**Additional file 9:** Five KEGG pathway maps of candidate genes in *Arabidopsis.* The enzymes whose genes are identified in *Arabidopsis* are shown by green-shaded rectangles, the rectangles with white background represent enzymes identified in other organisms but not currently linked to *Arabidopsis*. The green-shaded rectangles with red pentacle indicate that the genes have been mapped to the homologous genes underlying the QTL confidence interval and those genes are shown in red rectangles with arrows indicated (http://www.genome.jp/kegg/).
